# The C-Type Lectin Receptor CLECSF8/CLEC4D Is a Key Component of Anti-Mycobacterial Immunity

**DOI:** 10.1016/j.chom.2015.01.004

**Published:** 2015-02-11

**Authors:** Gillian J. Wilson, Mohlopheni J. Marakalala, Jennifer C. Hoving, Arjan van Laarhoven, Rebecca A. Drummond, Bernhard Kerscher, Roanne Keeton, Esther van de Vosse, Tom H.M. Ottenhoff, Theo S. Plantinga, Bachti Alisjahbana, Dhirendra Govender, Gurdyal S. Besra, Mihai G. Netea, Delyth M. Reid, Janet A. Willment, Muazzam Jacobs, Sho Yamasaki, Reinout van Crevel, Gordon D. Brown

**Affiliations:** 1 Institute of Medical Sciences, University of Aberdeen, Foresterhill, Aberdeen AB25 2ZD, UK; 2 Division of Immunology, Institute of Infectious Disease and Molecular Medicine, University of Cape Town, 792 Cape Town, South Africa; 3 Department of Internal Medicine, Radboud University Medical Center, Nijmegen 6525 GA, the Netherlands; 4 Department of Infectious Diseases, Leiden University Medical Center, Leiden 2333 ZA, the Netherlands; 5 Health Research Unit, Universitas Padjadjaran, Bandung 40161, Indonesia; 6 Division of Anatomical Pathology, University of Cape Town, 7925 Cape Town, South Africa; 7 School of Biosciences, University of Birmingham, Birmingham B15 2TT, UK; 8 Division of Molecular Immunology, Medical Institute of Bioregulation, Kyushu University, Fukuoka 108-8639, Japan

## Abstract

The interaction of microbes with pattern recognition receptors (PRRs) is essential for protective immunity. While many PRRs that recognize mycobacteria have been identified, none is essentially required for host defense in vivo. Here, we have identified the C-type lectin receptor CLECSF8 (CLEC4D, MCL) as a key molecule in anti-mycobacterial host defense. Clecsf8^−/−^ mice exhibit higher bacterial burdens and increased mortality upon *M. tuberculosis* infection. Additionally, Clecsf8 deficiency is associated with exacerbated pulmonary inflammation, characterized by enhanced neutrophil recruitment. Clecsf8^−/−^ mice show reduced mycobacterial uptake by pulmonary leukocytes, but infection with opsonized bacteria can restore this phagocytic defect as well as decrease bacterial burdens. Notably, a *CLECSF8* polymorphism identified in humans is associated with an increased susceptibility to pulmonary tuberculosis. We conclude that CLECSF8 plays a non-redundant role in anti-mycobacterial immunity in mouse and in man.

## Highlights

• Clecsf8 is required for anti-mycobacterial immunity
• Clecsf8 mediates non-opsonic mycobacterial recognition by pulmonary leukocytes
• Loss of Clecsf8 results in increased inflammation, bacterial burdens, and mortality
• A human *CLECSF8* polymorphism is associated with increased susceptibility to TB

## Graphical Abstract

**Figure:**
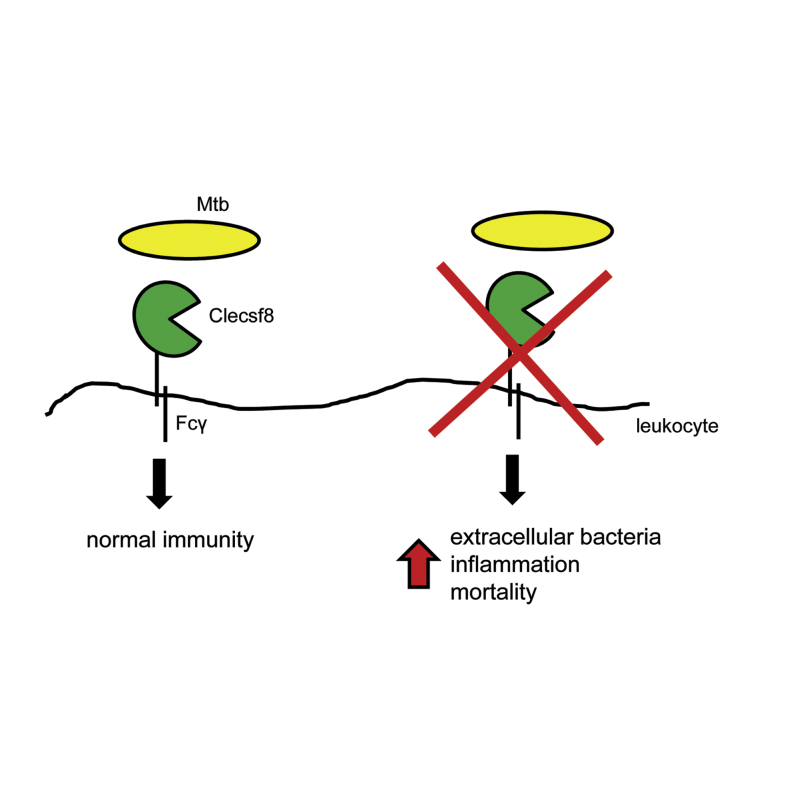

## Teaser

C-type lectin receptors (CLRs) are critical in anti-microbial host defense. Wilson et al. show that the CLR CLECSF8 is required for mycobacterial recognition by leukocytes. Loss of CLECSF8 results in neutrophilic inflammation, higher mycobacterial burdens, and increased mortality. Additionally, a *CLECSF8* polymorphism in humans is associated with susceptibility to tuberculosis.

## Introduction

Tuberculosis (TB) caused by *Mycobacterium tuberculosis* (Mtb) is one of the leading causes of infectious disease-related death worldwide. Mycobacterial recognition by innate immune cells is mediated by several pattern recognition receptors (PRRs), including members of the Toll-like receptor (TLR), NOD-like receptor (NLR), and C-type lectin receptor (CLR) families. These receptors activate inflammatory reactions that are essential for controlling the infection. Indeed, these early innate responses determine the outcome of disease and deficiencies in the major signaling adaptors downstream of these receptors, including MyD88 and Card9, rendering mice extremely susceptible to mycobacterial infection (Marakalala et al., 2011). Yet, despite convincing evidence from in vitro studies, no single PRR has yet been found to play a non-redundant role in anti-mycobacterial immunity in vivo (Marakalala et al., 2011). This has given rise to the assumption that recognition of *M. tuberculosis* involves multiple redundant interactions with numerous PRRs.

While the susceptibility of the MyD88-deficient mice to TB has been ascribed to defects in IL-1 receptor signaling (Fremond et al., 2007), the receptor(s) involved in the Card9-deficient phenotype has not been fully defined. Card9 is an essential component of the intracellular signaling pathway utilized by CLRs, and loss of this molecule leads to neutrophil-mediated pulmonary inflammation and rapid death in infected mice (Dorhoi et al., 2010). Three CLRs that utilize this pathway, Dectin-1, Mincle, and Dectin-2, have been described to recognize Mtb or its components. Dectin-1 was found to play a role in dendritic cell IL-12 production in response to mycobacteria in vitro; however, loss of this receptor did not alter susceptibility to infection in vivo (Marakalala et al., 2011). Mincle recognizes trehalose-6,6′-dimycolate (TDM or cord factor) and was found to mediate robust responses to this mycobacterial cell wall glycolipid both in vitro and in vivo (Ishikawa et al., 2009; Schoenen et al., 2010). However, the role of Mincle in vivo is controversial, with some studies describing no clear role for this receptor during mycobacterial infection (Behler et al., 2012; Heitmann et al., 2013). Dectin-2 induces pro- and anti-inflammatory cytokines in response to mannose-capped lipoarabinomannan, and knockout mice infected with *M. avium* presented with altered lung pathology at early time points during infection (Yonekawa et al., 2014). However, the importance of Dectin-2 during infection with Mtb is still unknown.

We recently identified another CLR (CLECSF8; CLEC4D) and have shown that it also recognizes TDM (Graham et al., 2012; Miyake et al., 2013). CLECSF8 is a member of the “Dectin-2 cluster” of CLRs and consists of a single extracellular C-type lectin-like domain, a stalk and transmembrane region, and a short cytoplasmic tail. The receptor is expressed by peripheral blood neutrophils, monocytes, and various subsets of dendritic cells (Graham et al., 2012). CLECSF8 can associate with FcRγ chain to trigger intracellular signaling, inducing phagocytosis, the respiratory burst, and the release of proinflammatory cytokines (Graham et al., 2012; Miyake et al., 2013). Moreover, like Mincle, Clecsf8 can drive both innate and adaptive immunity in response to TDM (Miyake et al., 2013). In this study, we have explored the role of Clecsf8 in vivo and have discovered that this CLR plays a non-redundant role in anti-mycobacterial immunity.

## Results

### Clecsf8 Is Required for Resistance to Mycobacterial Infection In Vivo

**Figure 1:**
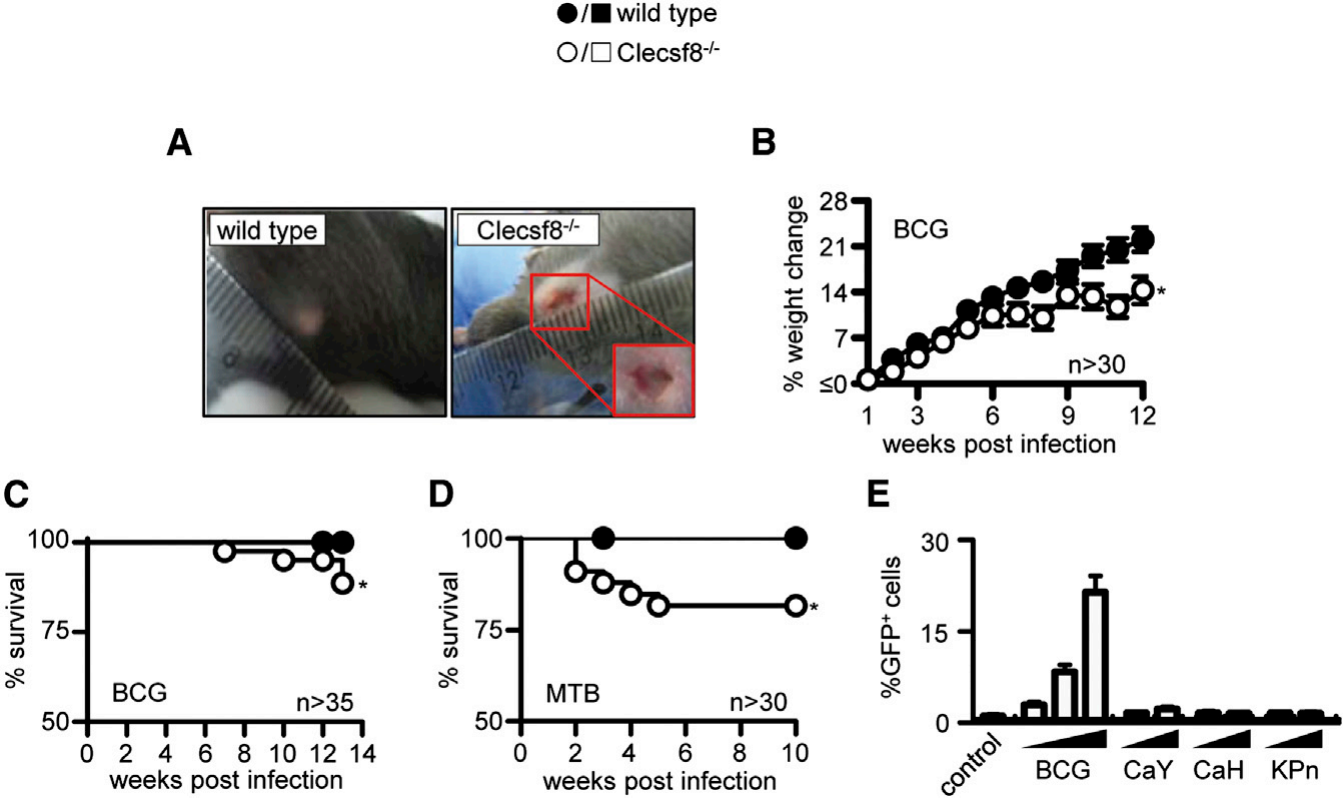
Clecsf8 Is Required for Resistance to Mycobacterial Infection In Vivo (A) Ulceration in Clecsf8^−/−^, but not wild-type (WT), mice at the site of injection with CFA. Change in weight (B; mean ± SEM) and survival curve (C) of Clecsf8^−/−^ and WT mice following i.t. infection with 5 × 10^5^*M. bovis* BCG. (D) Survival of WT and Clecsf8^−/−^ mice following aerosol infection with 100 CFU *M. tuberculosis* H37Rv. (E; mean ± SD) Analysis of GFP expression in Clecsf8-expressing reporter cells following stimulation with BCG (MOI: 1, 5, 15), *C. albicans* yeast (CaY; MOI: 5, 50), or hyphae (CaH; MOI: 5, 50), and *K. pneumoniae* (KPn; MOI: 5, 50), as indicated. Values in (B)–(D) are pooled data from at least two experiments, while the data in (E) are from one representative experiment. ^∗^p < 0.05. See also Figure S1.

We previously characterized the effect of Clecsf8 deficiency, but did not identify a role for this receptor in vivo, despite extensive analysis (Graham et al., 2012). However, during these experiments we noticed that subcutaneous immunization with complete Freund’s adjuvant (CFA) reproducibly led to ulceration at the injection site in more than 50% of the Clecsf8-deficient mice, an effect which was not apparent in the wild-type mice (Figure 1A; data not shown). Given that the major immune-stimulating component of CFA is Mtb, and that Clecsf8 can recognize TDM (Miyake et al., 2013), we investigated whether this receptor was required for anti-mycobacterial immunity in vivo.

We first determined whether the loss of Clecsf8 would influence the survival of mice during infection with live mycobacteria. In order to explore this possibility, wild-type and Clecsf8^−/−^ mice were challenged intra-tracheally (i.t.) with the attenuated vaccine strain *M. bovis* Bacille Calmette-Guerin (BCG), and survival of the animals was monitored over time. Notably, in contrast to the wild-type mice, the Clecsf8^−/−^ mice gained less weight (Figure 1B), and more than 10% of these animals succumbed to infection between 6 and 14 weeks (Figure 1C). Importantly, knockout mice aerosol infected with *M. tuberculosis* H37Rv also gained less weight, and 20% of these animals succumbed to the infection within 6 weeks (Figure 1D; data not shown). Longer-term experiments did not reveal any further reduction in survival of the Clecsf8-deficient mice compared to wild-type animals (data not shown).

Zhu and colleagues have recently suggested that Clecsf8 is also required for control of systemic infection with *Candida albicans* (Zhu et al., 2013), but only after low-dose infection. These results are in contrast to previous observations from several laboratories including our own (Graham et al., 2012), and repeated experiments using high and low doses of *C. albicans* failed to demonstrate any role for Clecsf8 in controlling this fungal pathogen (Figure S1A). Clecsf8 has also been implicated in immunity to *Klebsiella pneumoniae* (Steichen et al., 2013), but as with *Candida*, we observed no differences in mortality or weight loss in the knockout mice following i.t. infection with this organism (Figure S1B; data not shown). Importantly, *K. pneumoniae* and *C. albicans* both failed to stimulate GFP expression in Clecsf8-expressing reporter cells (Miyake et al., 2013), whereas these cells robustly induced GFP in response to BCG (Figure 1E).

Thus, these data identify Clecsf8 as a PRR with a non-redundant role in anti-mycobacterial immunity in vivo.

### Clecsf8 Is Not Required for Adaptive Responses to Mycobacteria

Purified ligands of many CLRs, including Clecsf8 (Miyake et al., 2013), can act as adjuvants and direct the development of adaptive immunity, but the role of these receptors in driving responses to intact microorganisms is less clear. Notably, acquired immunity to mycobacteria was unaffected by the loss of the major CLR intracellular signaling adaptor Card9 (Dorhoi et al., 2010). Nevertheless, we investigated the possibility that this receptor may be capable of modulating adaptive immunity using CFA as an adjuvant. However, no differences were observed in the Clecsf8^−/−^ mice in terms of the number, division, or activation of antigen-specific CD4^+^ T cells in the draining lymph nodes at the two time points that were examined post immunization (Figures S1C–S1F; data not shown). The knockout mice also developed normal antigen-specific immunoglobulin responses (Figure S1G). There were no defects in CD4/CD8 T cell ratios in the lungs during mycobacterial infection (Figure S1H). Clecsf8^−/−^ mice also displayed normal delayed-type hypersensitivity (Figure S1I) and mycobacterial-specific T cell recall responses (Figure S1J) following BCG vaccination. Thus, deficiency of Clecsf8 does not influence the development of acquired immunity to mycobacteria.

### Clecsf8 Is Involved in Controlling Bacterial Burdens, Cytokine Production, and Granuloma Formation In Vivo

**Figure 2:**
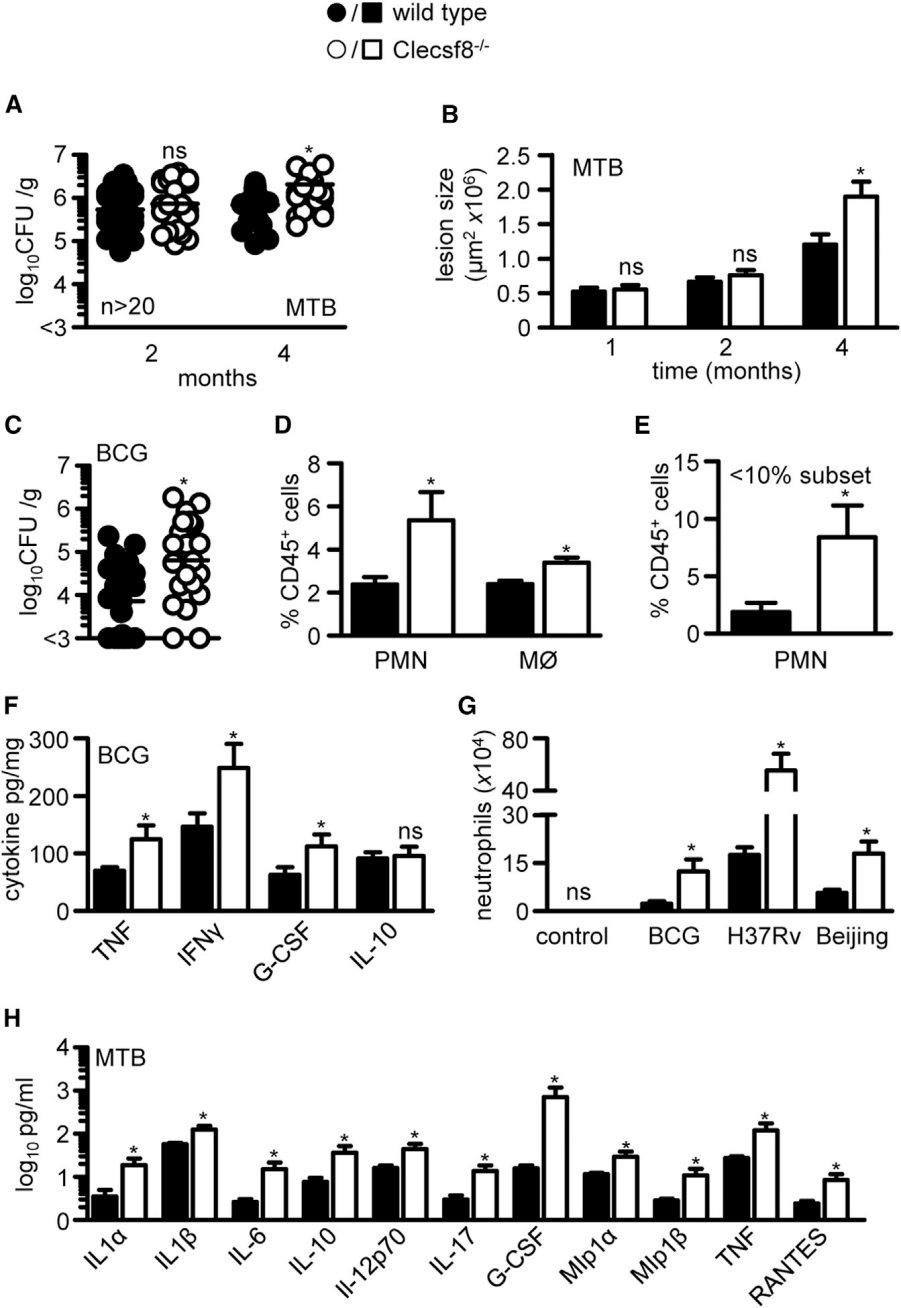
Clecsf8 Deficiency Results in Exacerbated Pulmonary Inflammation with Increased Accumulation of Neutrophils and Higher Bacterial Burdens (A) Pulmonary bacterial burdens in wild-type (WT) or Clecsf8^−/−^ mice following aerosol infection with 100 CFU *M. tuberculosis* H37Rv. (B) Pulmonary inflammatory lesion size over time. Pulmonary bacterial burdens (C) and leukocyte composition (D) in WT or Clecsf8^−/−^ mice 3 months following i.t. infection with 5 × 10^5^*M. bovis* BCG. (E) Neutrophil levels in WT (n = 3) and Clecsf8^−/−^ (n = 8) animals that show the greatest change in body weight (< 10%). (F) Pulmonary cytokine levels in 3-month *M. bovis* BCG-infected animals. (G) Pulmonary leukocyte composition in WT or Clecsf8^−/−^ mice 48 hr after i.t. infection with *M. bovis* BCG, *M. tuberculosis* H37Rv, or Beijing, as indicated. (H) BAL cytokine levels in mice at 48 hr after infection with *M. tuberculosis* H37Rv. Shown are pooled data (mean ± SEM). ^∗^p < 0.05. ns, not significant. See also Figure S2.

To examine how deficiency of Clecsf8 was affecting anti-mycobacterial immunity, we characterized the lungs of wild-type and Clecsf8^−/−^ mice following aerosol infection with *M. tuberculosis* H37Rv. At early time points after infection, we did not detect any difference in bacterial burdens, but by 4 months we observed moderately increased burdens in the infected knockout mice (∼0.50 log; Figure 2A). These increased bacterial burdens could be observed directly in Ziehl-Neelsen-stained tissue sections (Figure S2A), and analysis of the lungs of mice infected with Mtb revealed larger inflammatory lesions in the Clecsf8^−/−^ mice at later time points (Figures 2B and S2B). Similarly, increased bacterial burdens were also observed in BCG-infected knockout mice at later time points (Figure 2C), and cellular analysis of digested lung tissue at 3 months post infection revealed significantly more CD11b^+^Ly6G^high^ neutrophils and CD11b^+^F4/80^+^ macrophages in the Clecsf8^−/−^ mice (Figure 2D). Strikingly, Clecsf8^−/−^ mice most affected by infection, as determined by less than 10% weight gain, had the highest numbers of neutrophils in their lung, even when compared to wild-type mice with a similar phenotype (Figure 2E). Consistent with the increased cellular infiltrates, there were significantly higher levels of inflammatory cytokines, including TNF-α, IFN-γ, and G-CSF, in the lungs of the knockout mice (Figure 2F). There were no differences in IL-10 levels in the Clecsf8^−/−^ mice.

To gain further insights, we next characterized pulmonary inflammation 48 hr following the administration of a high dose of mycobacteria. Similar to the later time points, flow cytometry analysis and histology revealed a significant increase in neutrophils in the lungs of Clecsf8^−/−^ mice infected with either BCG, *M. tuberculosis* H37Rv, or the more pathogenic *M. tuberculosis* strain Beijing (Figures 2G and S2C). The cellular inflammatory response to *M. tuberculosis* H37Rv was accompanied by increased levels of many proinflammatory cytokines and chemokines, but also increased levels of IL-10 (Figure 2H). There were no differences in CFU recovered from wild-type and knockout mice at this early time point (Figure S2D). Therefore we conclude that deficiency of Clecsf8 results in higher mycobacterial burdens and increased pulmonary inflammation, which is predominantly neutrophilic.

### Clecsf8 Is Required for Mycobacterial Uptake

**Figure 3:**
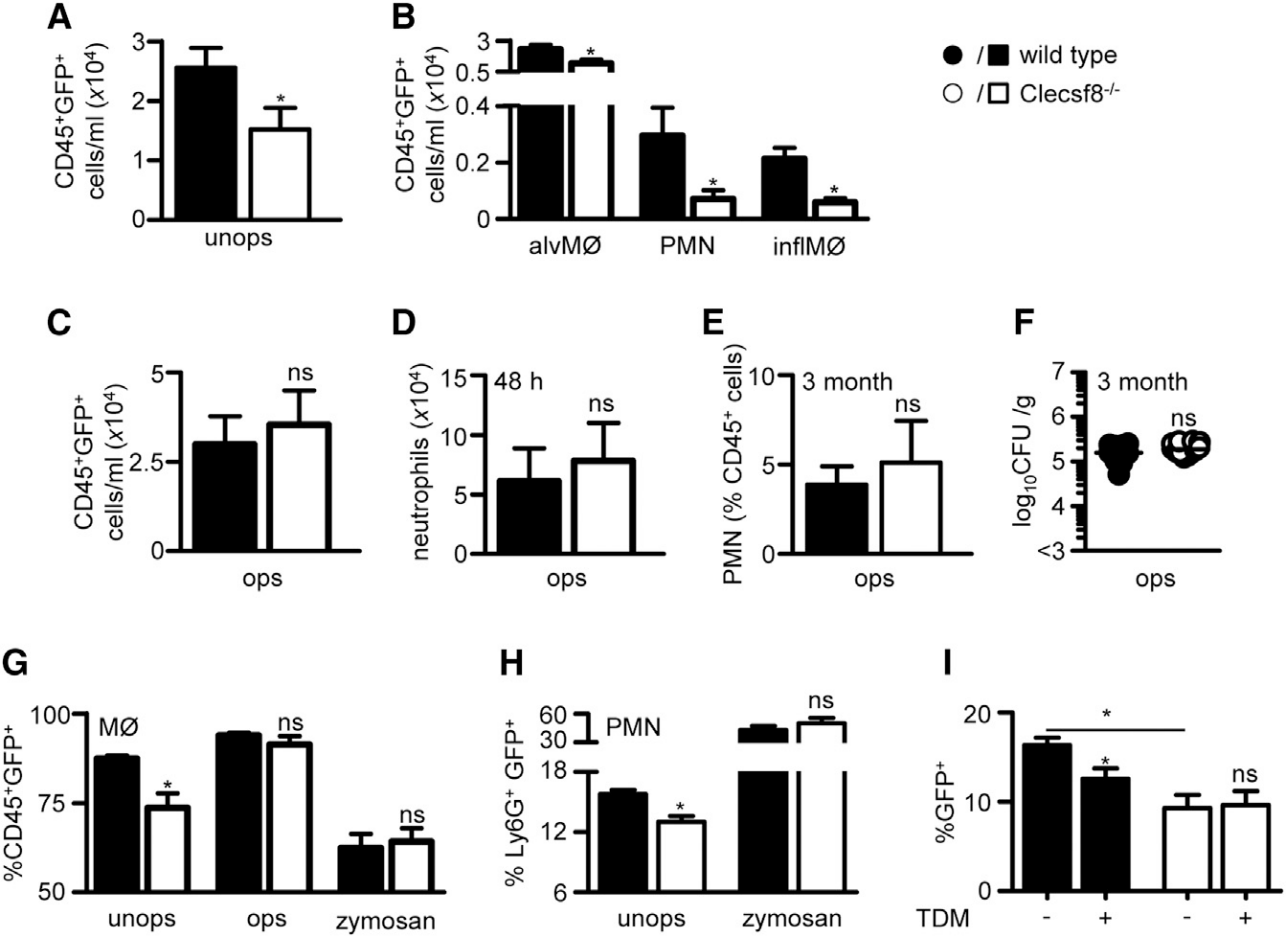
Clecsf8 Is Required for Mycobacterial Binding (A and B) Total GFP^+^ CD45^+^ cells (A) or particular cell types (B), as indicated, in the lungs of wild-type (WT) or Clecsf8^−/−^ mice 4 hr after infection with GFP-expressing *M. bovis* BCG (n > 14). (C and D) Total GFP^+^ CD45^+^ cells (C) and numbers of neutrophils (D) in BAL isolated from WT or Clecsf8^−/−^ mice after infection with opsonized *M. bovis* BCG at 4 and 48 hr, respectively (n > 10). (E and F) Numbers of neutrophils (E) and bacterial burdens (F) in the lungs of WT or Clecsf8^−/−^ mice 3 months after infection with opsonized *M. bovis* BCG (n = 12). (G and H) In vitro binding of unopsonized (unops) and opsonized (ops) GFP-expressing *M. bovis* BCG, or zymosan, to (G) thioglycollate-elicited macrophages or (H) neutrophils. (I) Effect of TDM on in vitro binding of GFP^+^BCG to thioglycollate-elicited macrophages isolated from WT or Clecsf8^−/−^ mice. Shown are pooled data (mean ± SEM) from at least two independent experiments. ^∗^p < 0.05. See also Figure S2.

We have previously shown that intracellular signaling from Clecsf8 can trigger particle phagocytosis (Graham et al., 2012; Miyake et al., 2013), and therefore examined the possibility that the phenotype of the Clecsf8^−/−^ mice was stemming from a defect in mycobacterial uptake and clearance by leukocytes. For these experiments, we infected mice with a GFP-expressing strain of *M. bovis* BCG and then characterized bacterial association with pulmonary CD45^+^ myeloid cells 4 hr after challenge. Notably, while the total number of pulmonary leukocytes was similar in both groups of mice at this early time point (Figure S2E), we observed significantly less mycobacterial association with leukocytes isolated from the Clecsf8^−/−^ mice, as determined by GFP positivity (Figure 3A). Characterization of these cells demonstrated defective mycobacterial association with all major pulmonary leukocyte subsets, including CD11c^+^SiglecF^+^ alveolar macrophages, CD11b^+^Ly6G^high^ neutrophils, and CD11b^+^F4/80^+^ macrophages (Figure 3B). Consistent with this observation, there were increased levels of non-cell-associated mycobacteria in the lungs of the Clecsf8^−/−^ mice (Figure S2F).

To demonstrate that the defect was solely due to loss of recognition by Clecsf8, we opsonized the bacteria with anti-BCG antibodies, prior to infection, and observed that association of the bacteria with leukocytes was fully restored in Clecsf8^−/−^ mice in vivo (Figure 3C). Unlike with unopsonized bacteria (Figure 2G), there was no difference in cellular inflammation at 48 hr in the knockout mice when challenged with opsonized bacteria (Figure 3D). Importantly, opsonization rescued the phenotype of the knockout mice even out to 3 months in terms of weight gain (Figure S2G), survival (Figure S2H), pulmonary neutrophil influx (Figure 3E), and bacterial burdens (Figure 3F).

We could also demonstrate defective mycobacterial association with Clecsf8^−/−^ thioglycollate-elicited macrophages (Figure 3G) and neutrophils (Figure 3H) in vitro. Clecsf8 deficiency specifically affected mycobacterial binding to leukocytes, but not phagocytosis, as the levels of ingestion of bacteria that were cell-bound was equivalent to wild-type cells (Figure S2I). Importantly, bacterial binding to knockout macrophages could be restored following opsonization, and was specific for mycobacteria as loss of Clecsf8 had no effect on association of the unrelated particle zymosan (Figure 3G). Moreover, we could show that TDM inhibited the binding of unopsonized mycobacteria with wild-type thioglycollate-elicited macrophages in vitro, but had no effect on bacterial binding to cells isolated from the knockout mice (Figure 3I). TDM had no effect on the association of zymosan with macrophages isolated from either strain of mice (Figure S2J). Thus, we conclude that the phenotype of the Clecsf8^−/−^ mice stems from defective mycobacterial recognition by leukocytes.

### Polymorphisms of Human CLECSF8 Cause Susceptibility to TB

To determine whether CLECSF8 may also be important for human anti-mycobacterial immunity, we examined publicly available micro-array data sets for effects of TB on the expression of this CLR. Expression of *CLECSF8* in whole blood was strongly upregulated in HIV-negative patients with pulmonary TB (PTB) compared to controls in five out of six cohorts from various geographic areas (Figure S3A). In mice, we observed similar increases in Clecsf8 expression on leukocytes during pulmonary infection (Figure S3B). In the UK TB cohort, expression data were also available for uninfected (tuberculin skin test-negative) and latently infected (tuberculin skin test-positive) controls; there was no difference in *CLECSF8* expression between these two groups (Figure S3C). Initiation of treatment in PTB patients led to normalization of *CLECSF8* expression over time (Figure S3D). The highest levels of expression of the receptor were observed in monocytes and neutrophils in peripheral blood, consistent with our earlier observations (Graham et al., 2012), and PTB was associated with significantly increased levels of expression on circulating neutrophils compared to healthy controls (Figure S3E). These differences cannot be explained by differences in leukocyte numbers, as absolute and relative neutrophil counts did not differ between active TB patients and controls (Berry et al., 2010).

As the expression of *CLECSF8* correlated with PTB, and as we had identified a role for this receptor in anti-mycobacterial immunity in mice, we then determined whether polymorphisms of this CLR had an influence on susceptibility to TB in humans. We genotyped three *CLECSF8* SNPs in a total of 1,000 confirmed PTB patients and 955 age- and gender-matched community controls from an Indonesian cohort collected in Jakarta and Bandung, West Java (Table S1). These SNPs were chosen as together they covered all haplotypes with a frequency of > 5%, as described in the HapMap database for Japanese and Han-Chinese populations (Table S1; Figure S3F). However, we found that the minor allele frequencies of the three *CLECSF8* SNPs were lower in the control Indonesian subjects than those described in the HapMap database (Tables 1 and S1).

Of the three polymorphisms, the combined GA and GG genotypes of the non-synonymous SNP rs4304840 were significantly associated with disease with an odds ratio (OR) of 1.33 with a 95% confidence interval of 1.02–1.73 (Figure S3F; Table 1). As the number of patients with the GG genotype was small, it seems likely that the G allele confers susceptibility in a dominant fashion. The functional relevance of the rs4304840 polymorphism is further demonstrated in available expression quantitative trait locus (eQTL) data, where we found the G allele to be significantly associated (p < 10^−4^) with altered *CLECSF8* expression (data not shown). The intronic SNP rs4486677, which showed a high degree of linkage disequilibrium with rs4304840 in HapMap, had a similar OR, which bordered significance (Figure S3F; Table 1). Haplotype analyses showed that the haplotypes with GG/GA alleles for rs4304840 had similar ORs, irrespective of the rs4486677 allele (data not shown). The SNP rs4883165, which is located 12 kb upstream of the *CLECSF8* gene, was not associated with disease (Figure S3F; Table 1). In conclusion, the GG and GA genotypes for *CLECSF8* rs4304840 are associated with susceptibility to PTB, irrespective of the genotype for the SNP rs4486677.

The rs4304840 polymorphism causes a non-synonymous change (Ser32Gly) in the transmembrane region of the protein (Graham et al., 2012). This change could influence the association of this CLR with the Fcγ adaptor and affect the ability of this receptor to be transported to the cell surface (Marakalala et al., 2011). To explore this, we generated constructs for both wild-type and mutated CLECSF8 and transfected them into fibroblasts. These experiments revealed that while both wild-type and mutated proteins were expressed at equivalent levels in transfected cells, there was a significant reduction in the surface expression of the mutated protein (Figure S3G). Thus the rs4304840 polymorphism reduces surface expression of CLECSF8.

## Discussion

CLRs have key functions in host defense, and although they are best known as PRRs for fungi, there is growing evidence that CLRs are also involved in host responses to mycobacteria (Marakalala et al., 2011). The most compelling data come from analysis of mice deficient in a central CLR-signaling adaptor, Card9, which were extremely susceptible to mycobacterial infection (Dorhoi et al., 2010). Yet despite the identification of several CLRs capable of mycobacterial recognition, all have been found to be dispensable during infection with Mtb in vivo (Marakalala et al., 2011). In this report, we identify the CLR Clecsf8 as a PRR with a non-redundant role in anti-mycobacterial immunity.

Loss of Clecsf8 led to exacerbated pulmonary inflammation, characterized by enhanced neutrophil recruitment and increased mycobacterial burdens, but had no effect on the development of adaptive immunity. This phenotype resembles that of the Card9^−/−^ mice; however, these animals presented with greater pathology, and all of the animals died shortly after infection, a severity that was linked to defects in IL-10 production (Dorhoi et al., 2010). Similar profound phenotypes have also been observed in mice lacking other essential immune components, such as IFNγ. In contrast, fewer Clecsf8^−/−^ mice succumbed to mycobacterial infection, and there was no loss of IL-10. This suggests that the levels of IL-10 were protecting the majority of the infected Clecsf8^−/−^ mice from lethal pathology, despite the enhanced inflammation and bacterial burdens that were present in their lungs.

This difference in phenotype raises the question about the relationship between Clecsf8 and Card9. Card9 is downstream of several PRRs implicated in mycobacterial recognition, including CLRs, NLRs, and TLRs, and deficiency of this adaptor is likely to affect all of these pathways. Yet mouse models have not revealed a clear role for any of the PRRs so far identified (Philips and Ernst, 2012). Although Clecsf8 has not formally been shown to require Card9, it triggers intracellular signaling via the Fcγ chain and Syk kinase, and therefore must utilize this pathway (Graham et al., 2012; Miyake et al., 2013). Clecsf8 also associates and functionally interacts with Dectin-2 (Zhu et al., 2013) and Mincle (Lobato-Pascual et al., 2013), both of which have also been implicated in anti-mycobacterial immunity (Ishikawa et al., 2009; Yonekawa et al., 2014). In fact, Clecsf8 stimulation is required for Mincle expression, at least in response to TDM (Miyake et al., 2013). However, we detected expression of both Dectin-2 and Mincle during mycobacterial infection in the Clecsf8^−/−^ mice (data not shown). Interestingly, expression of Clecsf8 with Fcγ alone was insufficient to mediate mycobacterial binding in transfected fibroblasts, suggesting that its ability to associate with these other receptors is an important component of its function (data not shown). Thus, despite the fact that these and other receptors are involved in mycobacterial recognition (mediating the IL-10 response discussed above, for example), Clecsf8 deficiency recapitulates the major components of the Card9^−/−^ phenotype.

In both the Card9^−/−^ (Dorhoi et al., 2010) and Clecsf8^−/−^ mice, pulmonary pathology was associated with an accumulation of neutrophils and higher levels of neutrophil-associated cytokines, such as G-CSF. Indeed, depletion of either neutrophils or G-CSF reduced inflammation and prolonged survival of the Card9^−/−^ mice (Dorhoi et al., 2010). However, the involvement of neutrophils during TB is still controversial, with evidence for both protective and non-protective roles during infection. In humans, infected neutrophils were found to predominate in the lungs of patients with active PTB, and a neutrophil-driven transcriptional signature in blood was shown to correlate with disease severity (Berry et al., 2010). Interestingly, even though lessening the clinical disease, depletion of neutrophils in the Card9^−/−^ mice did not affect bacterial burdens in the lung, demonstrating that these granulocytes were the major drivers of pathology and were not directly contributing to protective host responses (Dorhoi et al., 2010). Indeed, the ability of neutrophils to actually kill mycobacteria is also controversial (Lowe et al., 2012).

In humans, we show that neutrophils have the highest levels of *CLECSF8* expression (Graham et al., 2012). Importantly, we have identified the association of a polymorphism (rs4304840) in this receptor with increased susceptibility to PTB in an Indonesian cohort. This polymorphism causes a non-synonymous change (Ser32Gly) in the transmembrane region of the protein, which substantially reduces its expression at the cell surface. Genetic variations in several PRRs have been shown to influence mycobacterial disease susceptibility, severity, and/or outcome, but many of these observations have not been confirmed in other cohorts. Moreover, the effects of these PRR polymorphisms are also dependent on bacterial genotype (Caws et al., 2008). However, the involvement of Clecsf8 does not appear to be strain-specific, at least in our animal models (Figure 2G). Moreover, based on *M. tuberculosis* spoligotyping, we did not find any difference in allele frequency for rs4304840 (the non-synonymous SNP that showed an association with disease) between the cases infected by a Beijing strain (n = 182) versus those infected by other strains (n = 379) (p = 0.371; data not shown). It will be nevertheless important to validate our observations in additional patient cohorts and determine the effect, if any, of *CLECSF8* polymorphisms in other disease phenotypes, such as meningeal and pediatric TB.

Interestingly, the few families with mutations in Card9 have not been associated with an increased susceptibility to TB (Marakalala et al., 2011). While the underlying reasons for this are unclear, the intact adaptive responses (Dorhoi et al., 2010) may mediate protection due to successful vaccination of these patients in endemic areas. Another possible mitigating factor is the inability of human neutrophils to express IL-10 (Tamassia et al., 2013), one of the major defects causing the pathology in the Card9^−/−^ mice (Dorhoi et al., 2010). This suggests that the cellular functions of Card9 may differ in humans and mice during mycobacterial infection.

Neutrophils can internalize mycobacteria (Lowe et al., 2012), and we found that Clecsf8 deficiency resulted in defective mycobacterial association with these and several other leukocyte populations in the lung. Defective mycobacterial clearance in the Clecsf8-deficient mice led to increased levels of extracellular bacteria, exacerbating neutrophilic pulmonary inflammatory responses. In a small subset of infected knockout mice, these deregulated responses ultimately led to death. Restoring mycobacterial leukocyte association through antibody opsonization completely rescued the Clecsf8-deficient phenotype both in vitro and in vivo.

In addition to mycobacteria, Clecsf8 has been implicated in immunity to *Candida albicans* (Zhu et al., 2013) and *Klebsiella pneumoniae* (Steichen et al., 2013). Yet we found no defect in resistance to infection with either of these pathogens. The role of Clecsf8 in immunity to *C. albicans* is arguably the most controversial, as previous experiments (Graham et al., 2012; Lobato-Pascual et al., 2013) and the data shown here failed to show any role for this CLR in the control of this fungal pathogen. The underlying reasons for these disparate results remain to be determined.

Overall, our data show that mycobacterial recognition is the primary function of CLECSF8. Importantly, a polymorphism of CLECSF8 causing reduced surface expression associates with increased susceptibility to PTB in humans. In conclusion, CLECSF8 is a non-redundant component of anti-mycobacterial immunity.

## Experimental Procedures

### Animals

C57BL/6, Clecsf8^−/−^ (Graham et al., 2012), and OT.II mice (10–12 weeks old) were obtained from specific pathogen-free facilities at the University of Aberdeen (UoA) and University of Cape Town (UCT). Animal experiments were performed using age- and sex-matched mice and conformed to the animal care and welfare protocols approved by the UoA (project license 60/4007) and UCT (011/027 and 012/031).

### Strains, Growth Conditions, and Infections

*M. tuberculosis* strain H37Rv or Beijing and *M. bovis* BCG strain Pasteur were grown on Middlebrook 7H10 agar plates containing 10% ADC (BD Biosciences) or Middlebrook 7H9 broth containing 10% ADC and 0.05% Tween 80 (Sigma). GFP-expressing *M. bovis* BCG was cultured in the presence of 10 μg/ml kanamycin (Sigma). A total of 100 colony-forming units (CFU) of *M. tuberculosis* H37Rv was administered using an inhalation exposure system (Terre Haute). For i.t. inoculations, 5 × 10^5^ CFU *M. tuberculosis* or *M. bovis* BCG were administered to the caudal oropharynx of anesthetized mice. In some experiments, *M. bovis* BCG was opsonized with anti-BCG antiserum (Alpha Diagnostics) before i.t. challenge. Organs were homogenized in PBS containing 0.05% Triton X-100 and complete mini-EDTA-free protease inhibitors (Roche). Bacterial burdens were determined by plating onto Middlebrook 7H10 agar.

### Flow Cytometric Analysis of Lung Cells

Cells were obtained from the lung by bronchio-alveolar lavage (BAL) with PBS containing 5 mM EDTA (Gibco) or by enzymatic digest with DNase (Sigma-Aldrich) and liberase (Roche). Digested tissue was passed through 70-μm and 40-μm nylon filters, and erythrocytes were lysed in Pharm Lyse solution (BD Biosciences). The following antibodies were used: CD45.2, Ly6G, CD11c, CD11b, Siglec F, CD3, CD4, CD8, CD19, Vα2, CD45.1, CD62L, CD44, CD69, CD25, IFNγ, and F4/80 (BD Biosciences or AbD Serotec). FACS was performed using an LSRII, Fortessa, or FACSAria (BD Biosciences) and analyzed using FlowJo 7.6.4. Alveolar macrophages were defined as CD11c^+^ SiglecF^+^, neutrophils as CD11b^+^ Ly6G^high^, and macrophages as CD11b^high^ F4/80^+^.

### Cytokine Assays

Tissue homogenates (above) were centrifuged to remove debris and supernatant stored at −80°C. Cytokine levels were measured using the Bio-Plex Pro Mouse 23-Plex kit (Bio-Rad) or by ELISA (BD Biosciences OptEIA and R&D Systems). Cytokine levels of tissue homogenates were normalized to sample protein concentrations.

### Reporter Cell Analysis

Reporter cell analysis with NFAT-GFP expressing T hybridoma cells, co-transfected with mCLECSF8 and Fcγ, was performed as described previously (Miyake et al., 2013).

### BCG Binding Experiments

For in vivo binding experiments, 1.5 × 10^6^ CFU GFP-expressing *M. bovis* BCG was administered i.t., and BAL cells were isolated after 4 hr and analyzed by FACS. For in vitro binding experiments, BCG-GFP was added to thioglycollate-elicited macrophages (10:1) or neutrophils (1:1). In some experiments, TDM was added at 1 μg/ml. Cells were harvested and stained for CD45 and GFP positivity (indicating bacterial association) ascertained by FACS.

### Genotype Analysis and Ethics Statement

We made use of a cohort of PTB patients in Indonesia (see Supplemental Experimental Procedures). Peripheral blood samples and genotyping was performed as described previously (Songane et al., 2012). All individuals recruited had signed a written informed consent. The study protocol was approved by the review boards of the University of Indonesia, the Eijkman Institute for Molecular Biology, and the Medical Ethical Committee Arnhem-Nijmegen.

### Statistical Analysis

Data were analyzed using GraphPad Prism 5.04. Unpaired t test or non-parametric Mann-Whitney was applied for comparison of groups, as appropriate, and the Wilcoxon sign rank test for paired follow-up data. For genotyping analysis the Hardy-Weinberg equilibrium was checked for each SNP using the program HWE Version 1.10 (Rockefeller University). Significance was indicated by p < 0.05.

## Author Contributions

G.J.W. and J.C.H. performed experiments with BCG. M.J.M. and J.C.H. performed experiments with Mtb. A.v.L. performed the human studies.

## Figures and Tables

**Table 1:** Distribution of Polymorphism Allele and Genotype Frequencies in Cases and Controls

SNP	Allele or Genotype	Frequency in Cases (%)	Frequency in Controls (%)	p Value	OR (95% CI)	OR (95% CI)
rs4883165	T	1,896 (94.8%)	1,814 (95.0%)			
	G	104 (5.2%)	96 (5.0%)			
	TT	898 (89.8%)	861 (90.2%)	0.805	TT vs. TG & GG: 0.96 (0.72–1.29)	TT & TG vs. GG: 1.05 (0.15–7.45)
	TG	100 (10.0%)	92 (9.6%)			
	GG	2 (0.2%)	2 (0.2%)			
rs4304840	A	1,844 (92.3%)	1,795 (94.0%)			
	G	154 (7.7%)	115 (6.0%)			
	AA	849 (84.9%)	843 (88.3%)	0.037	AA vs. GA & GG: 1.33 (1.02–1.73)	AA & GA vs. GG: 1.28 (0.28–5.72)
	GA	146 (14.6%)	109 (11.4%)			
	GG	4 (0.4%)	3 (0.3%)			
rs4486677	T	1,927 (96.7%)	1,859 (97.5%)			
	G	65 (3.3%)	47 (2.5%)			
	TT	931 (93.5%)	906 (95.1%)	0.136	TT vs. TG: 1.35 (0.92–1.99)	TT & TG vs. GG: n/a
	TG	65 (6.5%)	47 (4.9%)			
	GG	0	0			

## Supplemental Information

Document S1. Figures S1–S3, Table S1, and Supplemental Experimental Procedures

Document S2. Article plus Supplemental Information
