# Supplementary material for: The C-Type Lectin Receptor CLECSF8/CLEC4D Is a Key Component of Anti-Mycobacterial Immunity
Source: Cell Host Microbe. 2015 Feb 11;17(2):252–9. doi: 10.1016/j.chom.2015.01.004 (PMC4334100; doi:10.1016/j.chom.2015.01.004)
Supplement: Document S1. Figures S1–S3, Table S1, and Supplemental Experimental Procedures [file mmc1.pdf]

Cell Host & Microbe, Volume *17*

Supplemental Information

The C-Type Lectin Receptor CLECSF8/CLEC4D Is

a Key Component of Anti-Mycobacterial Immunity

Gillian J. Wilson, Mohlopheni J. Marakalala, Jennifer C. Hoving, Arjan van Laarhoven, Rebecca A. Drummond, Bernhard Kerscher, Roanne Keeton, Esther van de Vosse, Tom H.M. Ottenhoff, Theo S. Plantinga, Bachti Alisjahbana, Dhirendra Govender, Gurdyal S. Besra, Mihai G. Netea, Delyth M. Reid, Janet A. Willment, Muazzam Jacobs, Sho Yamasaki, Reinout van Crevel, and Gordon D. Brown

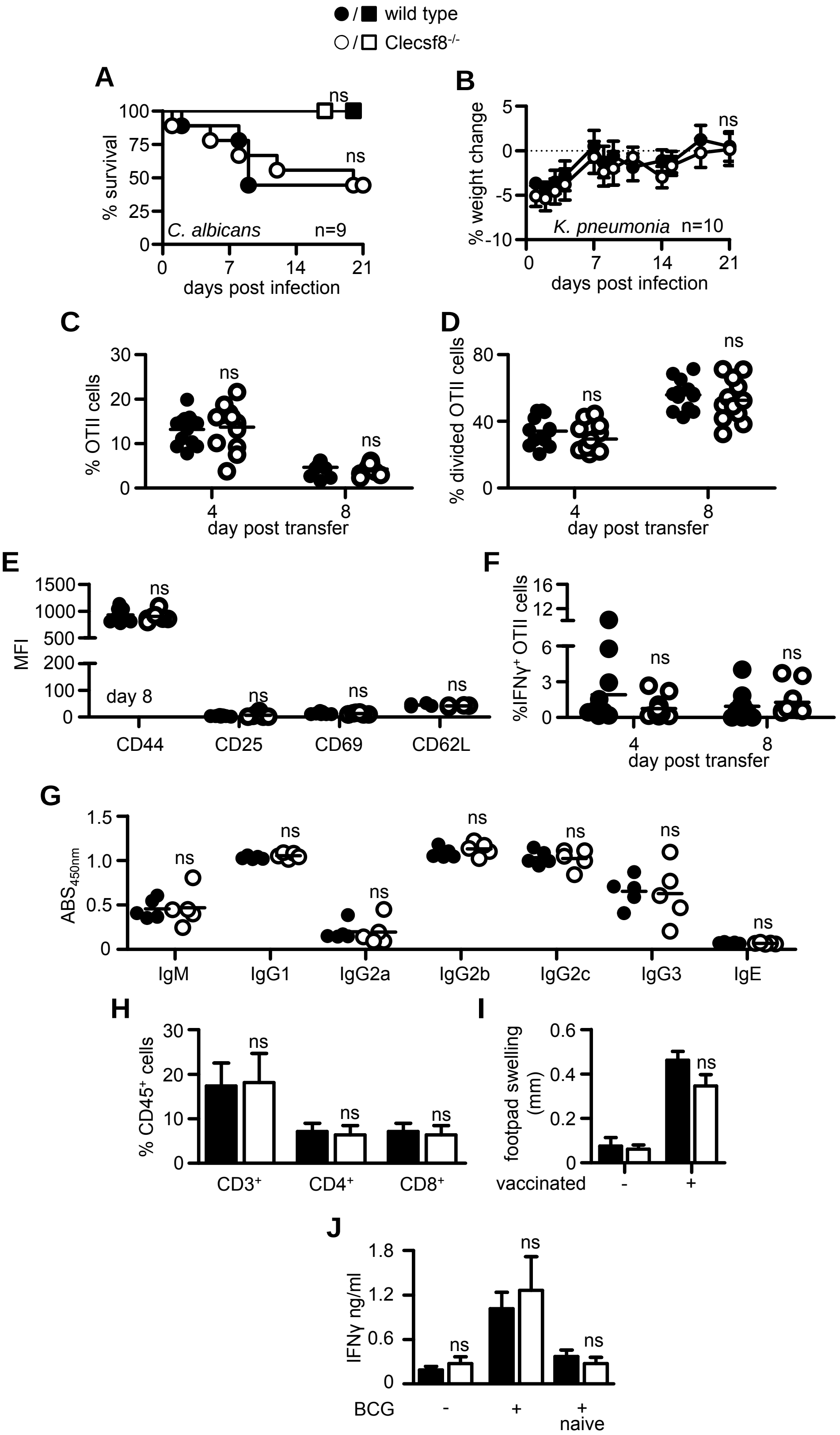

Wilson et al Supplemental Figure 1

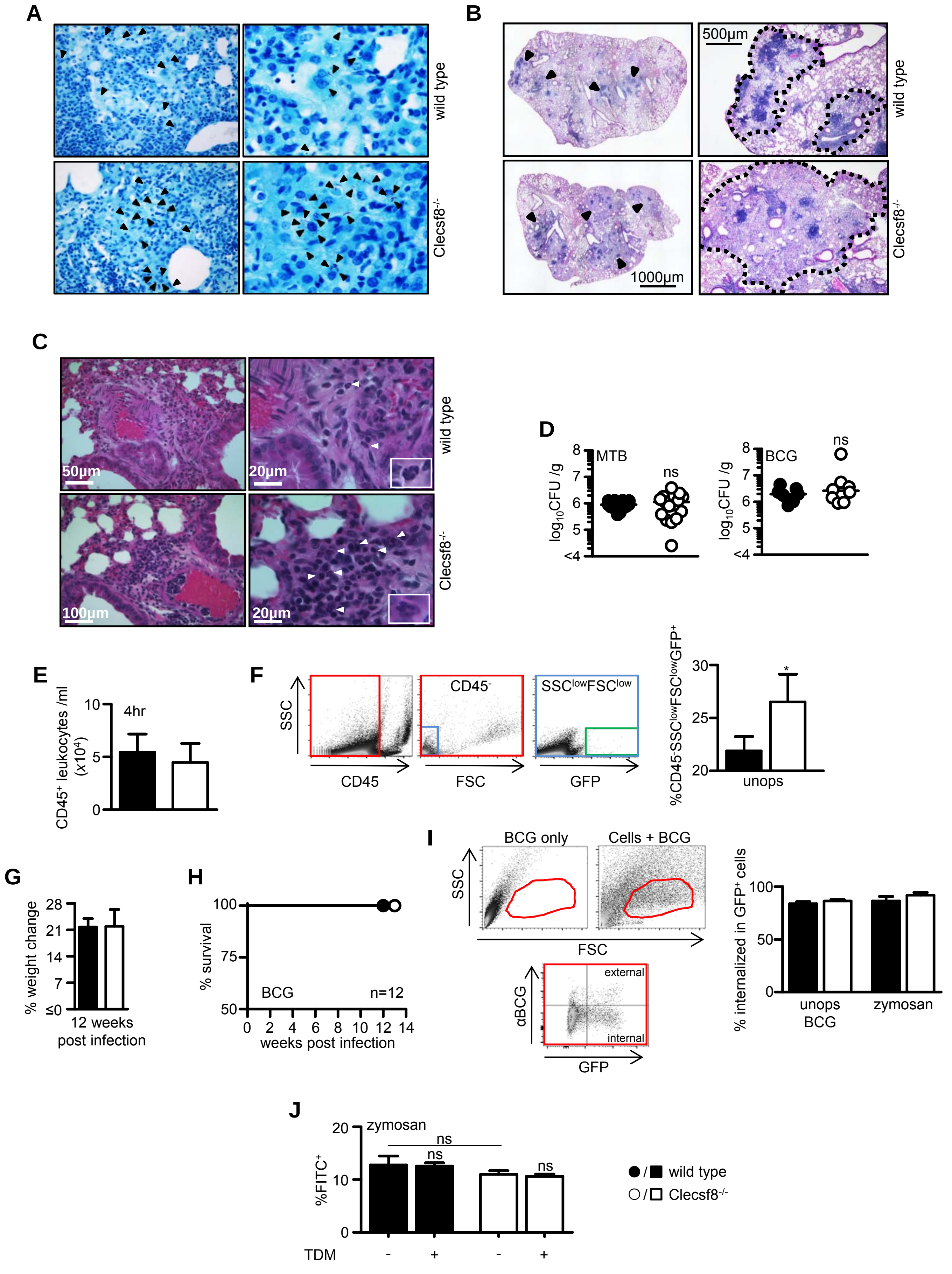

Wilson et al Supplemental Figure 2

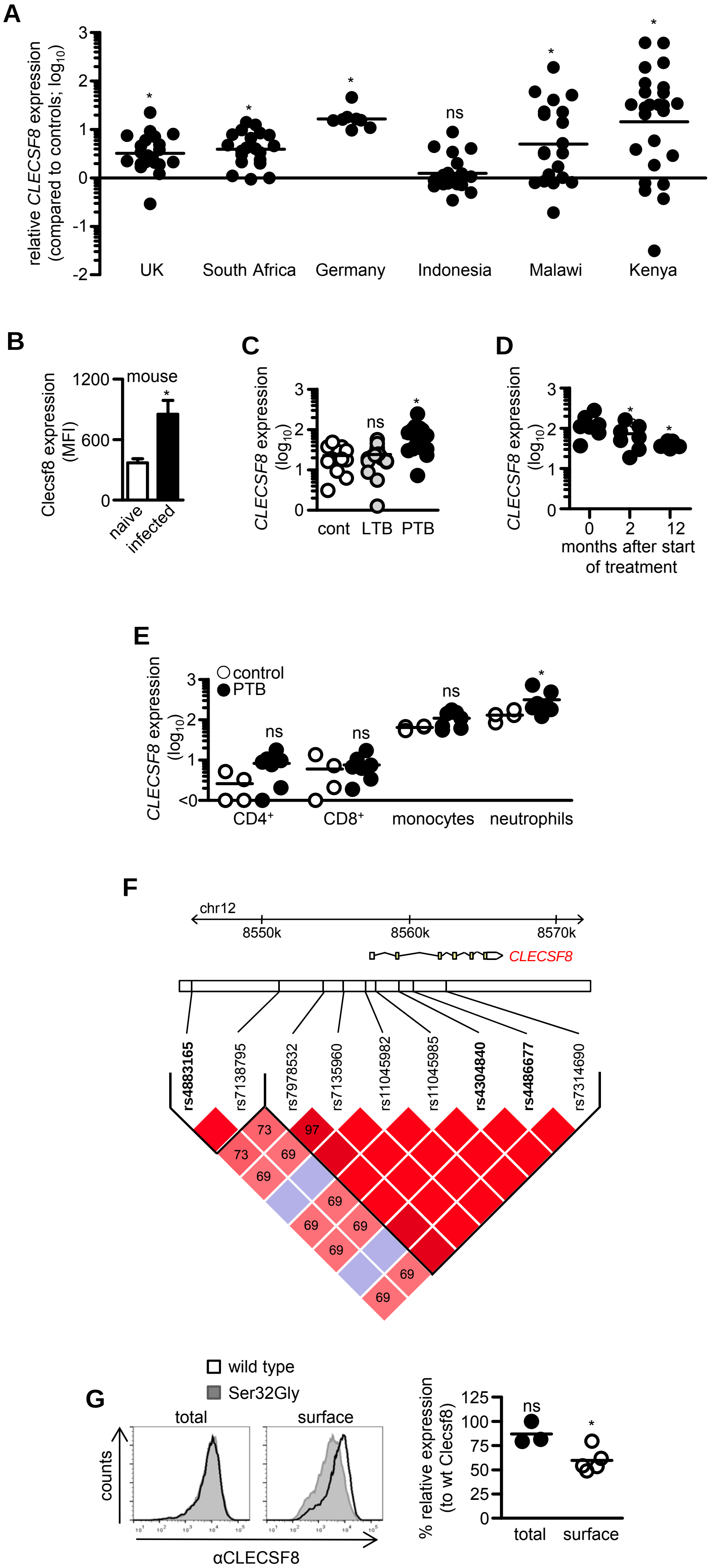

Wilson et al Supplemental Figure 3

SUPPLEMENTAL FIGURE LEGENDS

Figure S1: Clecsf8 is required for resistance to mycobacterial infection but does not impair adaptive immunity. Related to Figure 1. (A) Survival of wild-type and Clecsf8$^{-/-}$ following systemic (i.v.) infection with a high (2x10^5, circles) and a low (2x10^4, squares) dose of *Candida albicans* SC5314. (B) Change in weight of wild-type versus Clecsf8$^{-/-}$ mice following i.t. infection with 600 CFU *Klebsiella pneumoniae* strain 32. Characterization of the frequency (C), division (D), activation (E) and differentiation (F) of adoptively transferred OT.II T-cells in the draining lymph nodes of wild-type and Clecsf8$^{-/-}$ animals immunized with Ovalbumin and CFA. (G) Immunoglobulin responses in the serum of wild-type and Clecsf8$^{-/-}$ animals immunized with NP-KLH and CFA. (H) CD4$^+$ and CD8$^+$ T-cell populations in the lungs of wild-type and Clecsf8$^{-/-}$ mice 4 months after infection with 5x10^5 *M. bovis* BCG. (I) DTH responses in the footpad of wild-type and Clecsf8$^{-/-}$ mice 4 weeks after s.c. BCG vaccination. (J) IFN-γ recall responses following a 3-day BCG stimulation of lymph-node cells isolated from mice two weeks after infection. No cytokine response was obtained following stimulation of cells isolated from naïve animals (not shown). Values shown are from a representative experiment, except for (C, D, H - J) which are data from two pooled experiments. Each symbol (C to G) represents one mouse, and the data shown are the mean ± SD. *, $p < 0.05$; ns, not significant.

Figure S2: Clecsf8-deficiency results in higher bacterial burdens and exacerbated pulmonary inflammation. Related to Figure 2 and Figure3. (A) Ziehl-Neelsen stained lung sections from wild-type and Clecsf8$^{-/-}$ mice after 4 months of infection with *M. tuberculosis* H37Rv. Mycobacteria are indicated with arrows. (B)

Inflammatory lesion size 4 months after infection with *M. tuberculosis* H37Rv in H&E stained lung sections. (C) H&E stained lung sections at 48 hr showing accumulation of neutrophils (indicated with white arrows) after i.t. infection with a high dose ($5x10^5$) *M. tuberculosis* H37Rv. (D) Mycobacterial burdens in mice 48 h after i.t. infection with 5 x 10^5 CFU *M. tuberculosis* H37Rv or *M. bovis* BCG, as indicated. (E) Numbers of $CD45^+$ cells 4 h after i.t. inoculation of 1.5 x 10^6 CFU *M. bovis* BCG. (F) Gating strategy (control mouse shown for clarity) and percentage of non-cell associated mycobacteria (as detected in the green gate) in the lung 4 h after i.t. inoculation of 1.5 x 10^6 CFU GFP^+ *M. bovis* BCG. (G) Percentage weight change in mice 12 weeks after infection with opsonised *M. bovis* BCG. (H) Survival curve mice following i.t. infection with $5x10^5$ CFU of opsonised *M. bovis* BCG. (I) Gating strategy for flow cytometric assessment of levels of internalization of unopsonized BCG or zymosan bound to thioglycollate-elicited macrophages. Percentage internalized was defined as the percentage of APC^- (αBCG^-) GFP^+ or APC^- (FcDectin-1^-) $FITC^+$ cells versus total GFP^+ or $FITC^+$ cells. (J) Effect of TDM on *in vitro* binding of $FITC^+$ zymosan to isolated from wt or Clecsf8$^{-/-}$ mice. Shown in (D - I) are pooled data (mean ± SEM) from two independent experiments. *, $p < 0.05$.

Figure S3: Expression and polymorphisms of *CLECSF8.* Related to Table 1. (A) Relative expression of *CLECSF8* in whole blood in patients with active pulmonary TB of six cohorts, compared to the controls from the respective cohorts. (B) Clecsf8 expression on mouse $CD45^+$ leukocytes isolated from the lung 48 hr after BCG infection. (C) Expression of *CLECSF8* in whole blood in tuberculin skin test negative healthy control subjects (cont) from the UK, individuals with latent TB infection (LTB), and patients with active pulmonary TB (PTB). PTB and control data is the same as

UK cohort data in (A). (D) Expression of *CLECFS8* over time in PTB patients following initiation of TB treatment. (E) Expression of *CLECSF8* in $CD4^+$ and $CD8^+$ cells, monocytes and neutrophils of patients and healthy controls in the UK. *, $p < 0.05$. (F) Linkage disequilibrium plot of the *CLECSF8* gene region and 15kb upstream. SNPs with a prevalence of > 0.05 are shown with their linkage disequilibrium, based on data from the Han-Chinese (CHB) and Japanese (JPT) populations in Hapmap. The figure was generated with Haploview and then redrawn. Bright red indicates high level of linkage. The three SNPs that were investigated in this study (shown in bold) collectively tag the haplotypes that occur with a frequency of > 0.05 in CHB + JPT populations. (G) Example flow cytometry plots (left) and pooled analysis (right) of total and surface expression of mutated CLECSF8, relative to the wild type receptor in NIH3T3 cells expressing the Fcγ chain. Each cell line was independently generated and tested twice. *, $p < 0.05$; ns, not significant.

TABLE S1. The *CLECSF8* SNPs selected for analysis. Related to Table 1.

SNP	alleles	Minor allele frequency for the JPT & CHB population	Type
rs4883165	T / G	0.076	5'upstream of gene
rs4304840	A / G	0.129	nonsynonymous (Ser32Gly; transmembrane)
rs4486677	T / G	0.062	in intron 2

SUPPLEMENTAL EXPERIMENTAL PROCEDURES

Infections

Mice were challenged i.t. with 600 CFU *K. pneumonia* strain 32. Intravenous (i.v.) infection with low ($2x10^4$ CFU) and high ($2x10^5$ CFU) doses of *Candida albicans* SC5314 was carried out as described previously (Vautier et al., 2012). *C. albicans* hyphae were induced in 20% FCS in PBS for 3-5 h at 37°C.

Histology

Lungs were prepared for histology by fixing the large left lobe in 10% phosphate-buffered formalin and then embedding in paraffin. 5 µm-thick sections were stained with haematoxylin and eosin (H&E) for evaluation of pathologic changes and Ziehl-Neelsen (ZN) for *M. tuberculosis* detection. The sizes of all inflammatory lesions per section in infected mouse lungs were determined by automated morphometric analysis using a Nikon microscope eclipse 90i and the software NIS-Elements BR 3.1 (Nikon), as described previously (Schafer et al., 2009).

Adoptive Transfer

OT.II donor mice were culled and the lymph nodes and spleens were removed, disaggregated through 70 µM filters and white cells counted by trypan blue exclusion. $CD4^+$ cells were purified from single-cell suspensions by depleting irrelevant populations using biotin-antibody cocktail and anti-biotin micro-beads (Miltenyi Biotech). The purified $CD4^+$ cells were stained with 5 µM CFSE for 5 to 8 min at room temperature with continual rotation, and CFSE labelling was subsequently quenched by washing 2-3 times in 10% FCS in PBS. $CFSE^+CD4^+$ OT.II cells were checked by FACS for labelling efficiency and purity, which was

routinely >80%. $CFSE^+CD4^+$ OT.II cells were then injected intravenously into gender-matched recipient mice (3 to 5 x 10^6 cells per recipient) of the same background strain as the donors (C57BL/6). Mice were immunised 1 h following the adoptive transfer, with 50 µg purified ovalbumin (Hyglos GmBH) emulsified in Complete Freunds Adjuvant (Difco), delivered as 2 subcutaneous injections in the hind legs. Mice were sacrificed 4 and 8 days post-immunisation for analysis by flow cytometry. Inguinal (draining) lymph nodes were removed, made into single cell suspensions and processed for FACS, as described above standard methods. OT.II cells were defined as $CD4^+V\alpha2^+CD45.1^+$.

Immunoglobulin Assays

Mice were immunised with 100 µg NP-KLH (Biosearch Technologies) emulsified in CFA (Difco) delivered as three sub-cutaneous injections. Animals were sacrificed 10 days later, and anti-NP antibodies in the serum were measured by ELISA using specific secondary antibodies conjugated to HRP (Cambridge Biosciences).

BCG vaccination, DTH and recall responses

Mice were s.c. inoculated with 10^6 CFU *M. bovis* BCG strain Pasteur as previously described (Dorhoi et al., 2010). At 4 wk after vaccination, BCG-vaccinated and control mice were intradermally challenged with 2 µg PPD into the footpad. Footpad swelling was recorded 48 hr after challenge using a dial gauge caliper.

For recall responses, mice were infected i.t. with 5 x 10^5 CFU BCG. Draining lymph nodes were isolated after 2 weeks, disaggregated and plated at 5 x 10^5 cells per well in 96 well plates. Cells were then stimulated for 3 days with media only or

with a French-pressed BCG lysate. Cytokines were measured in the supernatant by ELISA (BD OptEIA).

Subject recruitment for genotype analysis

We previously recruited 1135 consecutive pulmonary TB patients diagnosed in two outpatient clinics and two hospitals in Jakarta and Bandung (Indonesia) from January 2001 to December 2006, for a series of genetic studies examining host susceptibility to TB ((Songane et al., 2012) and references therein). Diagnosis of pulmonary TB (PTB) was done according to WHO criteria by clinical presentation and chest radiograph examination, followed by confirmation with microscopic detection of acid-fast bacilli in ZN-stained sputum smears and positive culture of *M. tuberculosis* on 3% Ogawa medium. Patients with confirmed diagnosis of extra-pulmonary TB (n=93) and HIV-positive subjects (n=10) were excluded. During the same period, 1000 age and gender matched genetically unrelated community control subjects were selected, those with symptoms or chest X-rays suggesting possible active tuberculosis (n=48) were excluded from further analysis.

Genotyping and Selection of SNPs investigated

Using HaploView version 4.2 (Barrett et al., 2005) we accessed the publicly available HapMap Version 3 Release 2 to select SNPs for testing. As no data are available for the Indonesian population specifically, we based our selection on the data for the Han-Chinese (CHB) and Japanese (JPT) population. For *CLECSF8*, three SNPs tagged all the predicted haplotypes with frequencies of > 0.05 (See Figure S3 and Table S1).

In the current cohort, based on a disease prevalence of 262/100 000 (WHO Global tuberculosis report 2012), the power of detecting an allele that has a relative risk of 1.5 to disease, is 87% with a nominal significance of 0.05 (http://www.sph.umich.edu/csg/abecasis/cats/tour1.html).

Sample collection and genotype analysis

Peripheral blood samples were obtained by venapuncture. Genotyping was performed as described previously. In brief, genomic DNA was isolated from EDTA blood of patients and control subjects using standard methods. 5 ng of DNA was used for genotyping with multiplex assays designed using Mass ARRAY Designer Software (Sequenom) and genotypes were determined using Sequenom MALDI-TOF MS according to manufacturer's instructions (Sequenom Inc., San Diego, CA, USA). Briefly, the SNP region was amplified by a locus-specific PCR reaction. After amplification a single base extension from a primer adjacent to the SNP was performed to introduce mass differences between alleles. This was followed by salt removal and product spotting onto a target chip with 384 patches containing matrix. MALDI-TOF MS was then used to detect mass differences and genotypes were assigned real-time using Typer 4 software (Sequenom Inc. San Diego, CA, USA). As quality control, 5% of samples were genotyped in duplicate and each 384-well plate also contained at least 8 positive and 8 negative controls, no inconsistencies were observed. For quality control purposes the genotype of at least two samples for each homozygous genotype were confirmed by sequencing using Sanger method.

Human CLECSF8 Expression Analyses

Publicly available micro-array data sets from the Gene Expression Omnibus (GEO) were analysed for the transcript of *CLECSF8* in six cohorts. Expression in whole blood of HIV-negative pulmonary TB cases was compared to expression in healthy controls or latently infected individuals from the same setting with a Mann-Whitney U test. In order to display the expression of the different cohorts in one graph, data were log-transformed and then normalised by subtracting each value with the mean value of the controls of the respective cohort, divided by the standard deviation of the controls of the respective cohort. The UK cohort in the study of Berry et al., included data on follow-up, and on cell subsets ($CD4^+$ cells, $CD8^+$ cells, monocytes and neutrophils). The cohorts used were (country; patients; GEO accession number; assay; probe):

- UK and South-Africa; adult pulmonary TB cases; GSE19491; Illumina Human HT-12 V3 BeadChip; ILMN_1808979. (Berry et al., 2010).
- Germany; adult pulmonary TB cases; GSE34608; Agilent 4 × 44-k human expression arrays; A_23_P25235. (Maertzdorf et al., 2012).
- Indonesia; adult pulmonary TB cases; GSE56153; Illumina HumanRef-8 V3 BeadChip; ILMN_1808979. (Ottenhoff, 2012).
- Malawi and Kenya: paediatric TB cases; GSE39941; HumanHT-12 v.4 Expression BeadChip; ILMN_1808979. (Anderson et al., 2014).

For quantitative trait locus (eQTL) analysis of *CLECSF8* expression in whole blood, we used data available from a meta-analysis of >5000 individuals (online available at http://genenetwork.nl/bloodeqtlbrowser) (Westra et al., 2013) and the Genotype-Tissue Expression (GTEx) consortium (Consortium, 2013), which currently includes 168 individuals (online available at http://www.gtexportal.org/).

Phagocytosis assays

Phagocytosis was measured using our previously published methodology (Herre et al., 2004). In brief, thioglycollate elicited macrophages were incubated with BCG-GFP (10:1) or zymosan at 4 °C for 1 h to allow binding, washed and incubated at 37°C 5% for 3 h to allow uptake. Extracellular BCG-GFP was stained with primary anti-BCG rabbit antiserum (Alpha diagnostics) and secondary APC-conjugated goat anti-rabbit IgG (Life Technologies). Surface-bound FITC-labelled Zymosan was stained with biotinylated Fc-Dectin-1 (Graham et al., 2006) and streptavidin-APC (Invitrogen). Phagocytosis was defined as the percentage of GFP^+ or $FITC^+$ cells with internalised (APC^-) particles. Samples kept at 4^0C to prevent internalization were used as controls.

Generation of Cell lines

Generation of the pFBneo retroviral vector containing the full length hCLECSF8 open reading frame fused to an HA-tag was described previously (Graham et al., 2012). The S32G SNP (rs4304840) was introduced by a two-step PCR protocol. Firstly, the sequences downstream and upstream of the S32G SNP were amplified separately from pFB_hCLECSF8 using a vector specific and SNP-encoding primers (pFB-neo (5'-GCCAGGTTTCCGGGCCCTCAC-3') and hCLECSF8_S32G_F (5'-TAGTTTTCATCTTACTTCTCGGTGTCTGTTTTATTGCAAG-3'); pFB-retro (5'-GGCTGCCGACCCCGGGGGTGG-3') and hCLECSF8_S32G_R (5'-CTTGCAATAAAACAGACACCGAGAAGTAAGATGAAAACTA-3'). The original pFBneo_hCLECSF8 template was digested with DpnI followed by fusion and

amplification of the two PCR products using the vector specific primers. The PCR product was cloned into pFBneo_HA and the fidelity confirmed by sequencing.

NIH 3T3 cell lines stably co-expressing FcRγ with wild-type hCLECSF8-HA, hCLECSF8_S32G-HA or empty vector control were generated by retroviral transduction as described previously (Pyz and Brown, 2011). All cell lines were generated twice and used as non-clonal populations to reduce founder effects.

Receptor expression was assessed by flow cytometry as described in the main text. Briefly, cells were blocked in FACS wash (PBS, 5 mM EDTA, 0.5% BSA, 2 mM NaN_3) containing 5% heat-inactivated rabbit serum, followed by staining with an anti-HA antibody (HA.11, clone 16B12, Covance) for 1 h at 4°C. Cells were washed twice and stained with a goat-anti-mouse PE secondary antibody (Jackson) for 30 min at 4°C. After three additional washes, cells were fixed in 1% paraformaldehyde and analysed on a BD LSRII flow cytometer. To assess the total cellular CLECSF8 content, cells were fixed in 1% paraformaldehyde followed by permeabilisation with 0.5% saponin prior to assessing receptor expression, as described above. To calculate relative expression, mean fluorescence intensities (MFI) of all samples were normalised by subtracting their respective control background MFI. Subsequently, the MFI of each cell population was divided by the average MFI of wild-type hCLECSF8 expressing cells and expressed as a percentage. Data points plotted represent the means of independent experiments.

SUPPLEMENTAL REFERENCES

Anderson, S.T., Kaforou, M., Brent, A.J., Wright, V.J., Banwell, C.M., Chagaluka, G., Crampin, A.C., Dockrell, H.M., French, N., Hamilton, M.S., *et al.* (2014). Diagnosis of childhood tuberculosis and host RNA expression in Africa. N Engl J Med *370*, 1712-1723.

Barrett, J.C., Fry, B., Maller, J., and Daly, M.J. (2005). Haploview: analysis and visualization of LD and haplotype maps. Bioinformatics *21*, 263-265.

Berry, M.P., Graham, C.M., McNab, F.W., Xu, Z., Bloch, S.A., Oni, T., Wilkinson, K.A., Banchereau, R., Skinner, J., Wilkinson, R.J., *et al.* (2010). An interferon-inducible neutrophil-driven blood transcriptional signature in human tuberculosis. Nature *466*, 973-977.

Consortium, G.T. (2013). The Genotype-Tissue Expression (GTEx) project. Nat Genet *45*, 580-585.

Dorhoi, A., Desel, C., Yeremeev, V., Pradl, L., Brinkmann, V., Mollenkopf, H.J., Hanke, K., Gross, O., Ruland, J., and Kaufmann, S.H. (2010). The adaptor molecule CARD9 is essential for tuberculosis control. J Exp Med *207*, 777-792.

Graham, L.M., Gupta, V., Schafer, G., Reid, D.M., Kimberg, M., Dennehy, K.M., Horsnell, W.G., Guler, R., Campanero-Rhodes, M.A., Palma, A.S.*, et al.* (2012). The C-type lectin receptor CLECSF8(CLEC4D) is expressed by myeloid cells and triggers cellular activation through SYK kinase. J Biol Chem *287*, 25964-25974.

Graham, L.M., Tsoni, S.V., Willment, J.A., Williams, D.L., Taylor, P.R., Gordon, S., Dennehy, K., and Brown, G.D. (2006). Soluble Dectin-1 as a tool to detect beta-glucans. J Immunol Methods *314*, 164-169.

Herre, J., Marshall, A.J., Caron, E., Edwards, A.D., Williams, D.L., Schweighoffer, E., Tybulewicz, V.L., Reis e Sousa, C., Gordon, S., and Brown, G.D. (2004). Dectin-1 utilizes novel mechanisms for yeast phagocytosis in macrophages. Blood *104*, 4038-4045.

Maertzdorf, J., Weiner, J., 3rd, Mollenkopf, H.J., Bauer, T., Prasse, A., Muller-Quernheim, J., and Kaufmann, S.H. (2012). Common patterns and disease-related signatures in tuberculosis and sarcoidosis. Proc Natl Acad Sci U S A *109*, 7853-7858.

Ottenhoff, T.H. (2012). New pathways of protective and pathological host defense to mycobacteria. Trends Microbiol *20*, 419-428.

Pyz, E., and Brown, G.D. (2011). Screening for ligands of C-type lectin-like receptors. Methods Mol Biol *748*, 1-19.

Schafer, G., Guler, R., Murray, G., Brombacher, F., and Brown, G.D. (2009). The role of scavenger receptor B1 in infection with Mycobacterium tuberculosis in a murine model. PLoS ONE *4*, e8448.

Songane, M., Kleinnijenhuis, J., Alisjahbana, B., Sahiratmadja, E., Parwati, I., Oosting, M., Plantinga, T.S., Joosten, L.A., Netea, M.G., Ottenhoff, T.H.*, et al.* (2012). Polymorphisms in autophagy genes and susceptibility to tuberculosis. PLoS ONE *7*, e41618.

Vautier, S., Drummond, R.A., Redelinghuys, P., Murray, G.I., MacCallum, D.M., and Brown, G.D. (2012). Dectin-1 is not required for controlling Candida albicans colonization of the gastrointestinal tract. Infect Immun *80*, 4216-4222.

Westra, H.J., Peters, M.J., Esko, T., Yaghootkar, H., Schurmann, C., Kettunen, J., Christiansen, M.W., Fairfax, B.P., Schramm, K., Powell, J.E.*, et al.* (2013). Systematic identification of trans eQTLs as putative drivers of known disease associations. Nat Genet *45*, 1238-1243.
