# Supplementary material for: The C-Type Lectin Receptor CLECSF8/CLEC4D Is a Key Component of Anti-Mycobacterial Immunity
Source: Cell Host Microbe. 2015 Feb 11;17(2):252–9. doi: 10.1016/j.chom.2015.01.004 (PMC4334100; doi:10.1016/j.chom.2015.01.004)
Supplement: Document S2. Article plus Supplemental Information [file mmc2.pdf]

Short Article

Cell Host & Microbe

The C-Type Lectin Receptor CLECSF8/CLEC4D Is a Key Component of Anti-Mycobacterial Immunity

Graphical Abstract

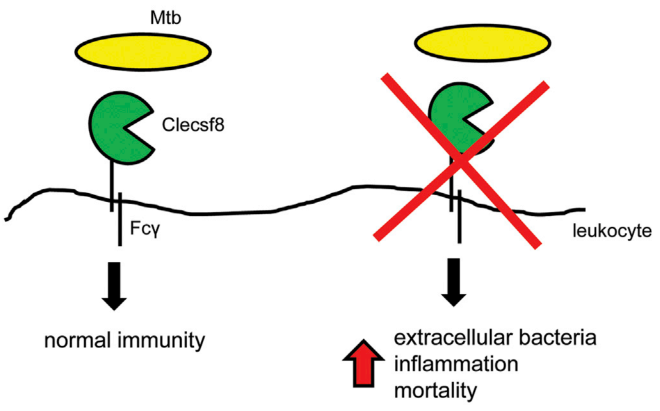

Highlights

- Clecsf8 is required for anti-mycobacterial immunity
- Clecsf8 mediates non-opsonic mycobacterial recognition by pulmonary leukocytes
- Loss of Clecsf8 results in increased inflammation, bacterial burdens, and mortality
- A human *CLECSF8* polymorphism is associated with increased susceptibility to TB

Authors

Gillian J. Wilson,
Mohlopheni J. Marakalala, ...,
Reinout van Crevel, Gordon D. Brown

Correspondence

gordon.brown@abdn.ac.uk

In Brief

C-type lectin receptors (CLRs) are critical in anti-microbial host defense. Wilson et al. show that the CLR CLECSF8 is required for mycobacterial recognition by leukocytes. Loss of CLECSF8 results in neutrophilic inflammation, higher mycobacterial burdens, and increased mortality. Additionally, a *CLECSF8* polymorphism in humans is associated with susceptibility to tuberculosis.

Wilson et al., 2015, Cell Host & Microbe *17*, 252–259
February 11, 2015 ©2015 The Authors
http://dx.doi.org/10.1016/j.chom.2015.01.004

Cell Host & Microbe
Short Article

The C-Type Lectin Receptor CLECSF8/CLEC4D Is a Key Component of Anti-Mycobacterial Immunity

Gillian J. Wilson,[1,9] Mohlopheni J. Marakalala,[2,9] Jennifer C. Hoving,[2,9] Arjan van Laarhoven,[3,9] Rebecca A. Drummond,[1] Bernhard Kerscher,[1] Roanne Keeton,[2] Esther van de Vosse,[4] Tom H.M. Ottenhoff,[4] Theo S. Plantinga,[3] Bachti Alisjahbana,[5] Dhirendra Govender,[6] Gurdyal S. Besra,[7] Mihai G. Netea,[3] Delyth M. Reid,[1] Janet A. Willment,[1] Muazzam Jacobs,[2] Sho Yamasaki,[8] Reinout van Crevel,[3] and Gordon D. Brown[1,2,*]
[1]Institute of Medical Sciences, University of Aberdeen, Foresterhill, Aberdeen AB25 2ZD, UK
[2]Division of Immunology, Institute of Infectious Disease and Molecular Medicine, University of Cape Town, 792 Cape Town, South Africa
[3]Department of Internal Medicine, Radboud University Medical Center, Nijmegen 6525 GA, the Netherlands
[4]Department of Infectious Diseases, Leiden University Medical Center, Leiden 2333 ZA, the Netherlands
[5]Health Research Unit, Universitas Padjadjaran, Bandung 40161, Indonesia
[6]Division of Anatomical Pathology, University of Cape Town, 7925 Cape Town, South Africa
[7]School of Biosciences, University of Birmingham, Birmingham B15 2TT, UK
[8]Division of Molecular Immunology, Medical Institute of Bioregulation, Kyushu University, Fukuoka 108-8639, Japan
[9]Co-first author
*Correspondence: gordon.brown@abdn.ac.uk
http://dx.doi.org/10.1016/j.chom.2015.01.004
This is an open access article under the CC BY license (http://creativecommons.org/licenses/by/4.0/).

SUMMARY

The interaction of microbes with pattern recognition receptors (PRRs) is essential for protective immunity. While many PRRs that recognize mycobacteria have been identified, none is essentially required for host defense in vivo. Here, we have identified the C-type lectin receptor CLECSF8 (CLEC4D, MCL) as a key molecule in anti-mycobacterial host defense. Clecsf8$^{-/-}$ mice exhibit higher bacterial burdens and increased mortality upon *M. tuberculosis* infection. Additionally, Clecsf8 deficiency is associated with exacerbated pulmonary inflammation, characterized by enhanced neutrophil recruitment. Clecsf8$^{-/-}$ mice show reduced mycobacterial uptake by pulmonary leukocytes, but infection with opsonized bacteria can restore this phagocytic defect as well as decrease bacterial burdens. Notably, a *CLECSF8* polymorphism identified in humans is associated with an increased susceptibility to pulmonary tuberculosis. We conclude that CLECSF8 plays a non-redundant role in anti-mycobacterial immunity in mouse and in man.

INTRODUCTION

Tuberculosis (TB) caused by *Mycobacterium tuberculosis* (Mtb) is one of the leading causes of infectious disease-related death worldwide. Mycobacterial recognition by innate immune cells is mediated by several pattern recognition receptors (PRRs), including members of the Toll-like receptor (TLR), NOD-like receptor (NLR), and C-type lectin receptor (CLR) families. These receptors activate inflammatory reactions that are essential for controlling the infection. Indeed, these early innate responses determine the outcome of disease and deficiencies in the major signaling adaptors downstream of these receptors, including MyD88 and Card9, rendering mice extremely susceptible to mycobacterial infection (Marakalala et al., 2011). Yet, despite convincing evidence from in vitro studies, no single PRR has yet been found to play a non-redundant role in anti-mycobacterial immunity in vivo (Marakalala et al., 2011). This has given rise to the assumption that recognition of *M. tuberculosis* involves multiple redundant interactions with numerous PRRs.

While the susceptibility of the MyD88-deficient mice to TB has been ascribed to defects in IL-1 receptor signaling (Fremond et al., 2007), the receptor(s) involved in the Card9-deficient phenotype has not been fully defined. Card9 is an essential component of the intracellular signaling pathway utilized by CLRs, and loss of this molecule leads to neutrophil-mediated pulmonary inflammation and rapid death in infected mice (Dorhoi et al., 2010). Three CLRs that utilize this pathway, Dectin-1, Mincle, and Dectin-2, have been described to recognize Mtb or its components. Dectin-1 was found to play a role in dendritic cell IL-12 production in response to mycobacteria in vitro; however, loss of this receptor did not alter susceptibility to infection in vivo (Marakalala et al., 2011). Mincle recognizes trehalose-6,6′-dimycolate (TDM or cord factor) and was found to mediate robust responses to this mycobacterial cell wall glycolipid both in vitro and in vivo (Ishikawa et al., 2009; Schoenen et al., 2010). However, the role of Mincle in vivo is controversial, with some studies describing no clear role for this receptor during mycobacterial infection (Behler et al., 2012; Heitmann et al., 2013). Dectin-2 induces pro- and anti-inflammatory cytokines in response to mannose-capped lipoarabinomannan, and knockout mice infected with *M. avium* presented with altered lung pathology at early time points during infection (Yonekawa et al., 2014). However, the importance of Dectin-2 during infection with Mtb is still unknown.

We recently identified another CLR (CLECSF8; CLEC4D) and have shown that it also recognizes TDM (Graham et al., 2012; Miyake et al., 2013). CLECSF8 is a member of the "Dectin-2

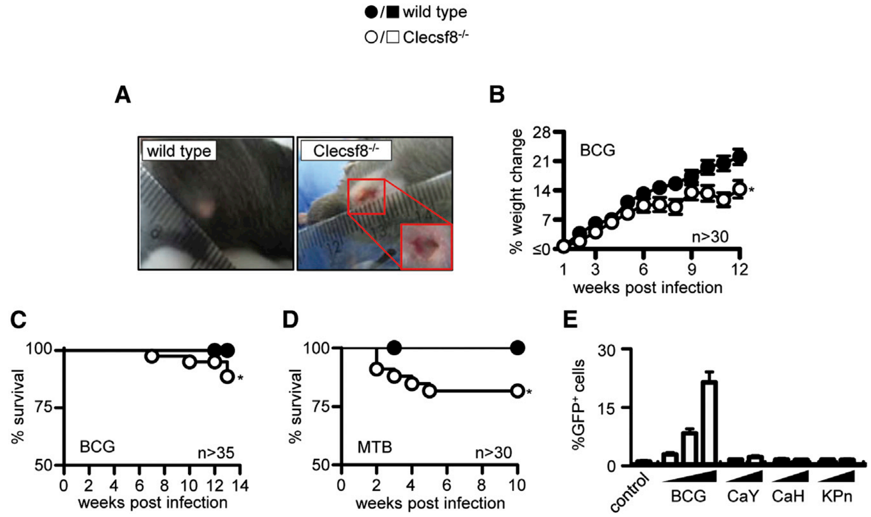

Figure 1. Clecsf8 Is Required for Resistance to Mycobacterial Infection In Vivo

(A) Ulceration in Clecsf8$^{-/-}$, but not wild-type (WT), mice at the site of injection with CFA. Change in weight (B; mean ± SEM) and survival curve (C) of Clecsf8$^{-/-}$ and WT mice following i.t. infection with 5×10^5 *M. bovis* BCG.

(D) Survival of WT and Clecsf8$^{-/-}$ mice following aerosol infection with 100 CFU *M. tuberculosis* H37Rv.

(E; mean ± SD) Analysis of GFP expression in Clecsf8-expressing reporter cells following stimulation with BCG (MOI: 1, 5, 15), *C. albicans* yeast (CaY; MOI: 5, 50), or hyphae (CaH; MOI: 5, 50), and *K. pneumoniae* (KPn; MOI: 5, 50), as indicated. Values in (B)–(D) are pooled data from at least two experiments, while the data in (E) are from one representative experiment. *$p < 0.05$. See also Figure S1.

cluster" of CLRs and consists of a single extracellular C-type lectin-like domain, a stalk and transmembrane region, and a short cytoplasmic tail. The receptor is expressed by peripheral blood neutrophils, monocytes, and various subsets of dendritic cells (Graham et al., 2012). CLECSF8 can associate with FcRγ chain to trigger intracellular signaling, inducing phagocytosis, the respiratory burst, and the release of proinflammatory cytokines (Graham et al., 2012; Miyake et al., 2013). Moreover, like Mincle, Clecsf8 can drive both innate and adaptive immunity in response to TDM (Miyake et al., 2013). In this study, we have explored the role of Clecsf8 in vivo and have discovered that this CLR plays a non-redundant role in anti-mycobacterial immunity.

RESULTS

Clecsf8 Is Required for Resistance to Mycobacterial Infection In Vivo

We previously characterized the effect of Clecsf8 deficiency, but did not identify a role for this receptor in vivo, despite extensive analysis (Graham et al., 2012). However, during these experiments we noticed that subcutaneous immunization with complete Freund's adjuvant (CFA) reproducibly led to ulceration at the injection site in more than 50% of the Clecsf8-deficient mice, an effect which was not apparent in the wild-type mice (Figure 1A; data not shown). Given that the major immune-stimulating component of CFA is Mtb, and that Clecsf8 can recognize TDM (Miyake et al., 2013), we investigated whether this receptor was required for anti-mycobacterial immunity in vivo.

We first determined whether the loss of Clecsf8 would influence the survival of mice during infection with live mycobacteria. In order to explore this possibility, wild-type and Clecsf8$^{-/-}$ mice were challenged intra-tracheally (i.t.) with the attenuated vaccine strain *M. bovis* Bacille Calmette-Guerin (BCG), and survival of the animals was monitored over time. Notably, in contrast to the wild-type mice, the Clecsf8$^{-/-}$ mice gained less weight (Figure 1B), and more than 10% of these animals succumbed to infection between 6 and 14 weeks (Figure 1C). Importantly, knockout mice aerosol infected with *M. tuberculosis* H37Rv also gained less weight, and 20% of these animals succumbed to the infection within 6 weeks (Figure 1D; data not shown). Longer-term experiments did not reveal any further reduction in survival of the Clecsf8-deficient mice compared to wild-type animals (data not shown).

Zhu and colleagues have recently suggested that Clecsf8 is also required for control of systemic infection with *Candida albicans* (Zhu et al., 2013), but only after low-dose infection. These results are in contrast to previous observations from several laboratories including our own (Graham et al., 2012), and repeated experiments using high and low doses of *C. albicans* failed to demonstrate any role for Clecsf8 in controlling this fungal pathogen (Figure S1A). Clecsf8 has also been implicated in immunity to *Klebsiella pneumoniae* (Steichen et al., 2013), but as with *Candida*, we observed no differences in mortality or weight loss in the knockout mice following i.t. infection with this organism (Figure S1B; data not shown). Importantly, *K. pneumoniae* and *C. albicans* both failed to stimulate GFP expression in Clecsf8-expressing reporter cells (Miyake et al., 2013), whereas these cells robustly induced GFP in response to BCG (Figure 1E).

Thus, these data identify Clecsf8 as a PRR with a non-redundant role in anti-mycobacterial immunity in vivo.

Clecsf8 Is Not Required for Adaptive Responses to Mycobacteria

Purified ligands of many CLRs, including Clecsf8 (Miyake et al., 2013), can act as adjuvants and direct the development of adaptive immunity, but the role of these receptors in driving responses to intact microorganisms is less clear. Notably, acquired immunity to mycobacteria was unaffected by the loss of the major CLR intracellular signaling adaptor Card9 (Dorhoi et al., 2010). Nevertheless, we investigated the possibility that this receptor may be capable of modulating adaptive immunity using CFA as an adjuvant. However, no differences were observed in the Clecsf8$^{-/-}$ mice in terms of the number, division, or activation of antigen-specific CD4$^+$ T cells in the draining lymph nodes at the two time points that were examined post immunization (Figures S1C–S1F; data not shown). The knockout mice also developed normal antigen-specific immunoglobulin responses (Figure S1G). There were no defects in CD4/CD8 T cell ratios in the lungs during mycobacterial infection (Figure S1H).

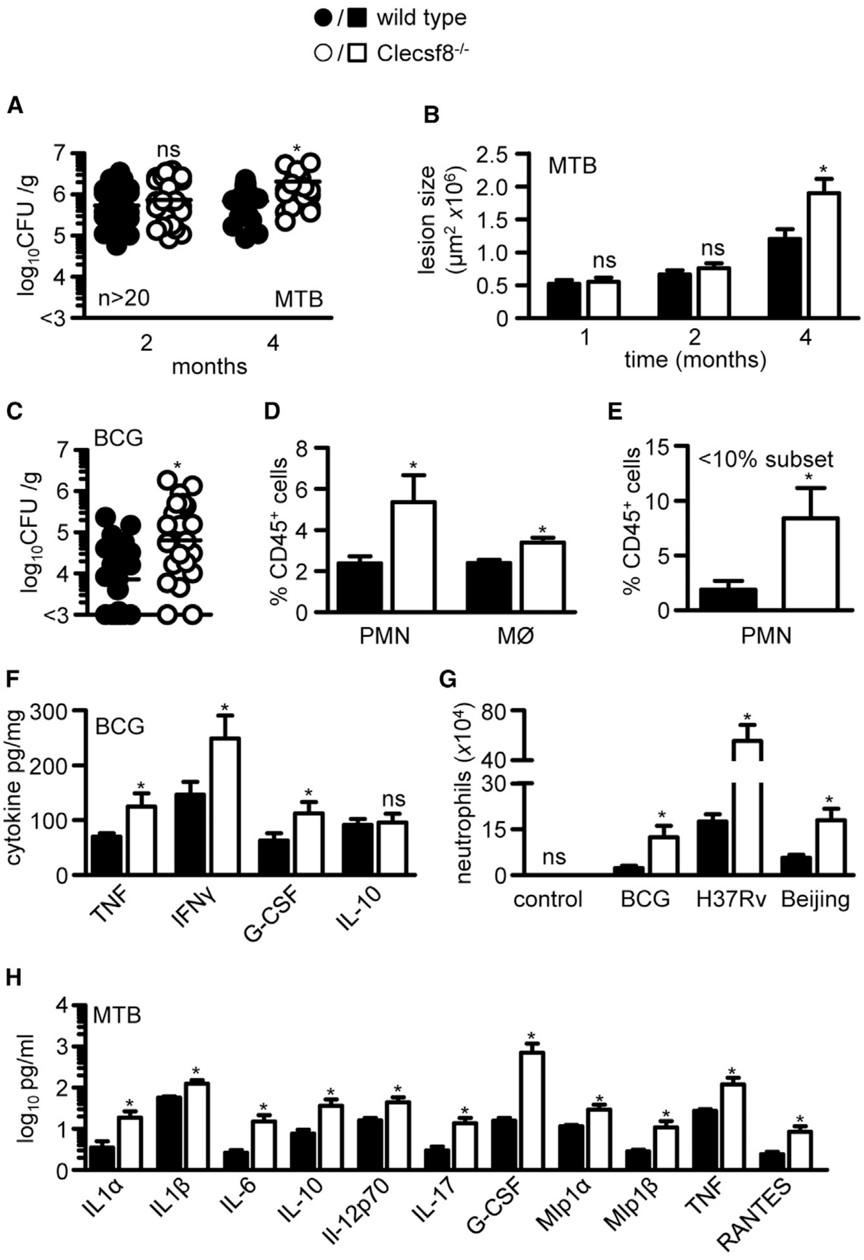

Figure 2. Clecsf8 Deficiency Results in Exacerbated Pulmonary Inflammation with Increased Accumulation of Neutrophils and Higher Bacterial Burdens

(A) Pulmonary bacterial burdens in wild-type (WT) or Clecsf8$^{-/-}$ mice following aerosol infection with 100 CFU *M. tuberculosis* H37Rv.

(B) Pulmonary inflammatory lesion size over time. Pulmonary bacterial burdens (C) and leukocyte composition (D) in WT or Clecsf8$^{-/-}$ mice 3 months following i.t. infection with 5×10^5 *M. bovis* BCG.

(E) Neutrophil levels in WT (n = 3) and Clecsf8$^{-/-}$ (n = 8) animals that show the greatest change in body weight (< 10%).

(F) Pulmonary cytokine levels in 3-month *M. bovis* BCG-infected animals.

(G) Pulmonary leukocyte composition in WT or Clecsf8$^{-/-}$ mice 48 hr after i.t. infection with *M. bovis* BCG, *M. tuberculosis* H37Rv, or Beijing, as indicated.

(H) BAL cytokine levels in mice at 48 hr after infection with *M. tuberculosis* H37Rv. Shown are pooled data (mean ± SEM). *$p < 0.05$. ns, not significant. See also Figure S2.

Clecsf8$^{-/-}$ mice also displayed normal delayed-type hypersensitivity (Figure S1I) and mycobacterial-specific T cell recall responses (Figure S1J) following BCG vaccination. Thus, deficiency of Clecsf8 does not influence the development of acquired immunity to mycobacteria.

Clecsf8 Is Involved in Controlling Bacterial Burdens, Cytokine Production, and Granuloma Formation In Vivo

To examine how deficiency of Clecsf8 was affecting anti-mycobacterial immunity, we characterized the lungs of wild-type and Clecsf8$^{-/-}$ mice following aerosol infection with *M. tuberculosis* H37Rv. At early time points after infection, we did not detect any difference in bacterial burdens, but by 4 months we observed moderately increased burdens in the infected knockout mice (~0.50 log; Figure 2A). These increased bacterial burdens could be observed directly in Ziehl-Neelsen-stained tissue sections (Figure S2A), and analysis of the lungs of mice infected with Mtb revealed larger inflammatory lesions in the Clecsf8$^{-/-}$ mice at later time points (Figures 2B and S2B). Similarly, increased bacterial burdens were also observed in BCG-infected knockout mice at later time points (Figure 2C), and cellular analysis of digested lung tissue at 3 months post infection revealed significantly more CD11b^{+}Ly6G^{high} neutrophils and CD11b^{+}F4/80^{+} macrophages in the Clecsf8$^{-/-}$ mice (Figure 2D). Strikingly, Clecsf8$^{-/-}$ mice most affected by infection, as determined by less than 10% weight gain, had the highest numbers of neutrophils in their lung, even when compared to wild-type mice with a similar phenotype (Figure 2E). Consistent with the increased cellular infiltrates, there were significantly higher levels of inflammatory cytokines, including TNF-α, IFN-γ, and G-CSF, in the lungs of the knockout mice (Figure 2F). There were no differences in IL-10 levels in the Clecsf8$^{-/-}$ mice.

To gain further insights, we next characterized pulmonary inflammation 48 hr following the administration of a high dose of mycobacteria. Similar to the later time points, flow cytometry analysis and histology revealed a significant increase in neutrophils in the lungs of Clecsf8$^{-/-}$ mice infected with either BCG, *M. tuberculosis* H37Rv, or the more pathogenic *M. tuberculosis* strain Beijing (Figures 2G and S2C). The cellular inflammatory response to *M. tuberculosis* H37Rv was

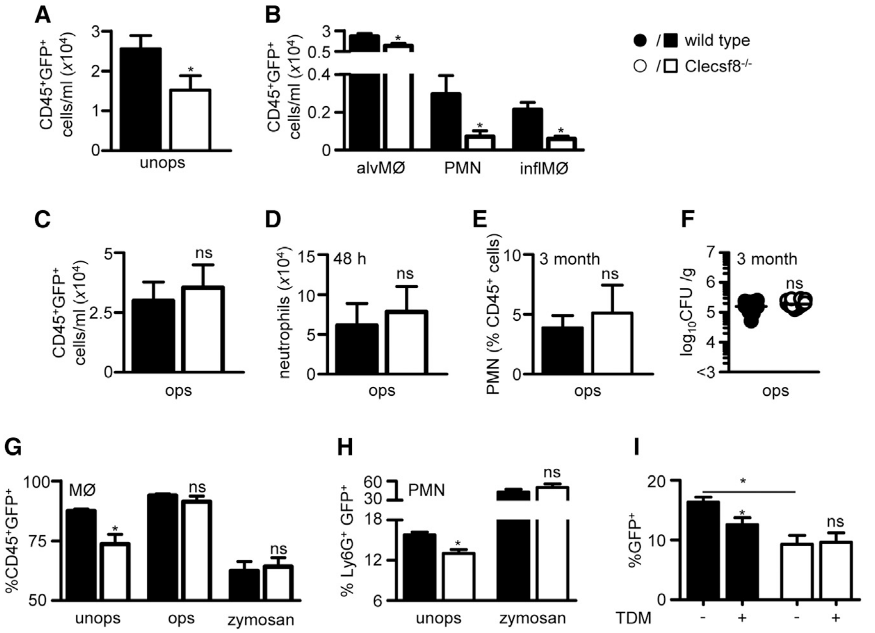

Figure 3. Clecsf8 Is Required for Mycobacterial Binding

(A and B) Total GFP$^+$ CD45$^+$ cells (A) or particular cell types (B), as indicated, in the lungs of wild-type (WT) or Clecsf8$^{-/-}$ mice 4 hr after infection with GFP-expressing *M. bovis* BCG (n > 14).

(C and D) Total GFP$^+$ CD45$^+$ cells (C) and numbers of neutrophils (D) in BAL isolated from WT or Clecsf8$^{-/-}$ mice after infection with opsonized *M. bovis* BCG at 4 and 48 hr, respectively (n > 10).

(E and F) Numbers of neutrophils (E) and bacterial burdens (F) in the lungs of WT or Clecsf8$^{-/-}$ mice 3 months after infection with opsonized *M. bovis* BCG (n = 12).

(G and H) In vitro binding of unopsonized (unops) and opsonized (ops) GFP-expressing *M. bovis* BCG, or zymosan, to (G) thioglycollate-elicited macrophages or (H) neutrophils.

(I) Effect of TDM on in vitro binding of GFP$^+$BCG to thioglycollate-elicited macrophages isolated from WT or Clecsf8$^{-/-}$ mice. Shown are pooled data (mean ± SEM) from at least two independent experiments. *p < 0.05. See also Figure S2.

accompanied by increased levels of many proinflammatory cytokines and chemokines, but also increased levels of IL-10 (Figure 2H). There were no differences in CFU recovered from wild-type and knockout mice at this early time point (Figure S2D). Therefore we conclude that deficiency of Clecsf8 results in higher mycobacterial burdens and increased pulmonary inflammation, which is predominantly neutrophilic.

Clecsf8 Is Required for Mycobacterial Uptake

We have previously shown that intracellular signaling from Clecsf8 can trigger particle phagocytosis (Graham et al., 2012; Miyake et al., 2013), and therefore examined the possibility that the phenotype of the Clecsf8$^{-/-}$ mice was stemming from a defect in mycobacterial uptake and clearance by leukocytes. For these experiments, we infected mice with a GFP-expressing strain of *M. bovis* BCG and then characterized bacterial association with pulmonary CD45$^+$ myeloid cells 4 hr after challenge. Notably, while the total number of pulmonary leukocytes was similar in both groups of mice at this early time point (Figure S2E), we observed significantly less mycobacterial association with leukocytes isolated from the Clecsf8$^{-/-}$ mice, as determined by GFP positivity (Figure 3A). Characterization of these cells demonstrated defective mycobacterial association with all major pulmonary leukocyte subsets, including CD11c$^+$SiglecF$^+$ alveolar macrophages, CD11b$^+$Ly6G^{high} neutrophils, and CD11b$^+$F4/80$^+$ macrophages (Figure 3B). Consistent with this observation, there were increased levels of non-cell-associated mycobacteria in the lungs of the Clecsf8$^{-/-}$ mice (Figure S2F).

To demonstrate that the defect was solely due to loss of recognition by Clecsf8, we opsonized the bacteria with anti-BCG antibodies, prior to infection, and observed that association of the bacteria with leukocytes was fully restored in Clecsf8$^{-/-}$ mice in vivo (Figure 3C). Unlike with unopsonized bacteria (Figure 2G), there was no difference in cellular inflammation at 48 hr in the knockout mice when challenged with opsonized bacteria (Figure 3D). Importantly, opsonization rescued the phenotype of the knockout mice even out to 3 months in terms of weight gain (Figure S2G), survival (Figure S2H), pulmonary neutrophil influx (Figure 3E), and bacterial burdens (Figure 3F).

We could also demonstrate defective mycobacterial association with Clecsf8$^{-/-}$ thioglycollate-elicited macrophages (Figure 3G) and neutrophils (Figure 3H) in vitro. Clecsf8 deficiency specifically affected mycobacterial binding to leukocytes, but not phagocytosis, as the levels of ingestion of bacteria that were cell-bound was equivalent to wild-type cells (Figure S2I). Importantly, bacterial binding to knockout macrophages could be restored following opsonization, and was specific for mycobacteria as loss of Clecsf8 had no effect on association of the unrelated particle zymosan (Figure 3G). Moreover, we could show that TDM inhibited the binding of unopsonized mycobacteria with wild-type thioglycollate-elicited macrophages in vitro, but had no effect on bacterial binding to cells isolated from the knockout mice (Figure 3I). TDM had no effect on the association of zymosan with macrophages isolated from either strain of mice (Figure S2J). Thus, we conclude that the phenotype of the Clecsf8$^{-/-}$ mice stems from defective mycobacterial recognition by leukocytes.

Polymorphisms of Human CLECSF8 Cause Susceptibility to TB

To determine whether CLECSF8 may also be important for human anti-mycobacterial immunity, we examined publicly available micro-array data sets for effects of TB on the expression of this CLR. Expression of *CLECSF8* in whole blood was strongly upregulated in HIV-negative patients with pulmonary TB (PTB) compared to controls in five out of six cohorts from various geographic areas (Figure S3A). In mice, we observed similar increases in Clecsf8 expression on leukocytes during pulmonary infection (Figure S3B). In the UK TB cohort, expression data were also available for uninfected (tuberculin skin test-negative) and latently infected (tuberculin skin test-positive) controls; there was no difference in *CLECSF8* expression between these two

Table 1. Distribution of Polymorphism Allele and Genotype Frequencies in Cases and Controls

SNP	Allele or Genotype	Frequency in Cases (%)	Frequency in Controls (%)	p Value	OR (95% CI)	OR (95% CI)
rs4883165	T	1,896 (94.8%)	1,814 (95.0%)			
	G	104 (5.2%)	96 (5.0%)			
	TT	898 (89.8%)	861 (90.2%)	0.805	TT vs. TG & GG: 0.96 (0.72–1.29)	TT & TG vs. GG: 1.05 (0.15–7.45)
	TG	100 (10.0%)	92 (9.6%)			
	GG	2 (0.2%)	2 (0.2%)			
rs4304840	A	1,844 (92.3%)	1,795 (94.0%)			
	G	154 (7.7%)	115 (6.0%)			
	AA	849 (84.9%)	843 (88.3%)	0.037	AA vs. GA & GG: 1.33 (1.02–1.73)	AA & GA vs. GG: 1.28 (0.28–5.72)
	GA	146 (14.6%)	109 (11.4%)			
	GG	4 (0.4%)	3 (0.3%)			
rs4486677	T	1,927 (96.7%)	1,859 (97.5%)			
	G	65 (3.3%)	47 (2.5%)			
	TT	931 (93.5%)	906 (95.1%)	0.136	TT vs. TG: 1.35 (0.92–1.99)	TT & TG vs. GG: n/a
	TG	65 (6.5%)	47 (4.9%)			
	GG	0	0			

groups (Figure S3C). Initiation of treatment in PTB patients led to normalization of *CLECSF8* expression over time (Figure S3D). The highest levels of expression of the receptor were observed in monocytes and neutrophils in peripheral blood, consistent with our earlier observations (Graham et al., 2012), and PTB was associated with significantly increased levels of expression on circulating neutrophils compared to healthy controls (Figure S3E). These differences cannot be explained by differences in leukocyte numbers, as absolute and relative neutrophil counts did not differ between active TB patients and controls (Berry et al., 2010).

As the expression of *CLECSF8* correlated with PTB, and as we had identified a role for this receptor in anti-mycobacterial immunity in mice, we then determined whether polymorphisms of this CLR had an influence on susceptibility to TB in humans. We genotyped three *CLECSF8* SNPs in a total of 1,000 confirmed PTB patients and 955 age- and gender-matched community controls from an Indonesian cohort collected in Jakarta and Bandung, West Java (Table S1). These SNPs were chosen as together they covered all haplotypes with a frequency of > 5%, as described in the HapMap database for Japanese and Han-Chinese populations (Table S1; Figure S3F). However, we found that the minor allele frequencies of the three *CLECSF8* SNPs were lower in the control Indonesian subjects than those described in the HapMap database (Tables 1 and S1).

Of the three polymorphisms, the combined GA and GG genotypes of the non-synonymous SNP rs4304840 were significantly associated with disease with an odds ratio (OR) of 1.33 with a 95% confidence interval of 1.02–1.73 (Figure S3F; Table 1). As the number of patients with the GG genotype was small, it seems likely that the G allele confers susceptibility in a dominant fashion. The functional relevance of the rs4304840 polymorphism is further demonstrated in available expression quantitative trait locus (eQTL) data, where we found the G allele to be significantly associated ($p < 10^{-4}$) with altered *CLECSF8* expression (data not shown). The intronic SNP rs4486677, which showed a high degree of linkage disequilibrium with rs4304840 in HapMap, had a similar OR, which bordered significance (Figure S3F; Table 1). Haplotype analyses showed that the haplotypes with GG/GA alleles for rs4304840 had similar ORs, irrespective of the rs4486677 allele (data not shown). The SNP rs4883165, which is located 12 kb upstream of the *CLECSF8* gene, was not associated with disease (Figure S3F; Table 1). In conclusion, the GG and GA genotypes for *CLECSF8* rs4304840 are associated with susceptibility to PTB, irrespective of the genotype for the SNP rs4486677.

The rs4304840 polymorphism causes a non-synonymous change (Ser32Gly) in the transmembrane region of the protein (Graham et al., 2012). This change could influence the association of this CLR with the Fcγ adaptor and affect the ability of this receptor to be transported to the cell surface (Marakalala et al., 2011). To explore this, we generated constructs for both wild-type and mutated CLECSF8 and transfected them into fibroblasts. These experiments revealed that while both wild-type and mutated proteins were expressed at equivalent levels in transfected cells, there was a significant reduction in the surface expression of the mutated protein (Figure S3G). Thus the rs4304840 polymorphism reduces surface expression of CLECSF8.

DISCUSSION

CLRs have key functions in host defense, and although they are best known as PRRs for fungi, there is growing evidence that CLRs are also involved in host responses to mycobacteria (Marakalala et al., 2011). The most compelling data come from analysis of mice deficient in a central CLR-signaling adaptor, Card9, which were extremely susceptible to mycobacterial infection

(Dorhoi et al., 2010). Yet despite the identification of several CLRs capable of mycobacterial recognition, all have been found to be dispensable during infection with Mtb in vivo (Marakalala et al., 2011). In this report, we identify the CLR Clecsf8 as a PRR with a non-redundant role in anti-mycobacterial immunity.

Loss of Clecsf8 led to exacerbated pulmonary inflammation, characterized by enhanced neutrophil recruitment and increased mycobacterial burdens, but had no effect on the development of adaptive immunity. This phenotype resembles that of the Card9$^{-/-}$ mice; however, these animals presented with greater pathology, and all of the animals died shortly after infection, a severity that was linked to defects in IL-10 production (Dorhoi et al., 2010). Similar profound phenotypes have also been observed in mice lacking other essential immune components, such as IFNγ. In contrast, fewer Clecsf8$^{-/-}$ mice succumbed to mycobacterial infection, and there was no loss of IL-10. This suggests that the levels of IL-10 were protecting the majority of the infected Clecsf8$^{-/-}$ mice from lethal pathology, despite the enhanced inflammation and bacterial burdens that were present in their lungs.

This difference in phenotype raises the question about the relationship between Clecsf8 and Card9. Card9 is downstream of several PRRs implicated in mycobacterial recognition, including CLRs, NLRs, and TLRs, and deficiency of this adaptor is likely to affect all of these pathways. Yet mouse models have not revealed a clear role for any of the PRRs so far identified (Philips and Ernst, 2012). Although Clecsf8 has not formally been shown to require Card9, it triggers intracellular signaling via the Fcγ chain and Syk kinase, and therefore must utilize this pathway (Graham et al., 2012; Miyake et al., 2013). Clecsf8 also associates and functionally interacts with Dectin-2 (Zhu et al., 2013) and Mincle (Lobato-Pascual et al., 2013), both of which have also been implicated in anti-mycobacterial immunity (Ishikawa et al., 2009; Yonekawa et al., 2014). In fact, Clecsf8 stimulation is required for Mincle expression, at least in response to TDM (Miyake et al., 2013). However, we detected expression of both Dectin-2 and Mincle during mycobacterial infection in the Clecsf8$^{-/-}$ mice (data not shown). Interestingly, expression of Clecsf8 with Fcγ alone was insufficient to mediate mycobacterial binding in transfected fibroblasts, suggesting that its ability to associate with these other receptors is an important component of its function (data not shown). Thus, despite the fact that these and other receptors are involved in mycobacterial recognition (mediating the IL-10 response discussed above, for example), Clecsf8 deficiency recapitulates the major components of the Card9$^{-/-}$ phenotype.

In both the Card9$^{-/-}$ (Dorhoi et al., 2010) and Clecsf8$^{-/-}$ mice, pulmonary pathology was associated with an accumulation of neutrophils and higher levels of neutrophil-associated cytokines, such as G-CSF. Indeed, depletion of either neutrophils or G-CSF reduced inflammation and prolonged survival of the Card9$^{-/-}$ mice (Dorhoi et al., 2010). However, the involvement of neutrophils during TB is still controversial, with evidence for both protective and non-protective roles during infection. In humans, infected neutrophils were found to predominate in the lungs of patients with active PTB, and a neutrophil-driven transcriptional signature in blood was shown to correlate with disease severity (Berry et al., 2010). Interestingly, even though lessening the clinical disease, depletion of neutrophils in the Card9$^{-/-}$ mice did not affect bacterial burdens in the lung, demonstrating that these granulocytes were the major drivers of pathology and were not directly contributing to protective host responses (Dorhoi et al., 2010). Indeed, the ability of neutrophils to actually kill mycobacteria is also controversial (Lowe et al., 2012).

In humans, we show that neutrophils have the highest levels of *CLECSF8* expression (Graham et al., 2012). Importantly, we have identified the association of a polymorphism (rs4304840) in this receptor with increased susceptibility to PTB in an Indonesian cohort. This polymorphism causes a non-synonymous change (Ser32Gly) in the transmembrane region of the protein, which substantially reduces its expression at the cell surface. Genetic variations in several PRRs have been shown to influence mycobacterial disease susceptibility, severity, and/or outcome, but many of these observations have not been confirmed in other cohorts. Moreover, the effects of these PRR polymorphisms are also dependent on bacterial genotype (Caws et al., 2008). However, the involvement of Clecsf8 does not appear to be strain-specific, at least in our animal models (Figure 2G). Moreover, based on *M. tuberculosis* spoligotyping, we did not find any difference in allele frequency for rs4304840 (the non-synonymous SNP that showed an association with disease) between the cases infected by a Beijing strain (n = 182) versus those infected by other strains (n = 379) ($p = 0.371$; data not shown). It will be nevertheless important to validate our observations in additional patient cohorts and determine the effect, if any, of *CLECSF8* polymorphisms in other disease phenotypes, such as meningeal and pediatric TB.

Interestingly, the few families with mutations in Card9 have not been associated with an increased susceptibility to TB (Marakalala et al., 2011). While the underlying reasons for this are unclear, the intact adaptive responses (Dorhoi et al., 2010) may mediate protection due to successful vaccination of these patients in endemic areas. Another possible mitigating factor is the inability of human neutrophils to express IL-10 (Tamassia et al., 2013), one of the major defects causing the pathology in the Card9$^{-/-}$ mice (Dorhoi et al., 2010). This suggests that the cellular functions of Card9 may differ in humans and mice during mycobacterial infection.

Neutrophils can internalize mycobacteria (Lowe et al., 2012), and we found that Clecsf8 deficiency resulted in defective mycobacterial association with these and several other leukocyte populations in the lung. Defective mycobacterial clearance in the Clecsf8-deficient mice led to increased levels of extracellular bacteria, exacerbating neutrophilic pulmonary inflammatory responses. In a small subset of infected knockout mice, these deregulated responses ultimately led to death. Restoring mycobacterial leukocyte association through antibody opsonization completely rescued the Clecsf8-deficient phenotype both in vitro and in vivo.

In addition to mycobacteria, Clecsf8 has been implicated in immunity to *Candida albicans* (Zhu et al., 2013) and *Klebsiella pneumoniae* (Steichen et al., 2013). Yet we found no defect in resistance to infection with either of these pathogens. The role of Clecsf8 in immunity to *C. albicans* is arguably the most controversial, as previous experiments (Graham et al., 2012; Lobato-Pascual et al., 2013) and the data shown here failed to show any role for this CLR in the control of this fungal pathogen. The underlying reasons for these disparate results remain to be determined.

Overall, our data show that mycobacterial recognition is the primary function of CLECSF8. Importantly, a polymorphism of CLECSF8 causing reduced surface expression associates with increased susceptibility to PTB in humans. In conclusion, CLECSF8 is a non-redundant component of anti-mycobacterial immunity.

EXPERIMENTAL PROCEDURES

Animals

C57BL/6, Clecsf8$^{-/-}$ (Graham et al., 2012), and OT.II mice (10–12 weeks old) were obtained from specific pathogen-free facilities at the University of Aberdeen (UoA) and University of Cape Town (UCT). Animal experiments were performed using age- and sex-matched mice and conformed to the animal care and welfare protocols approved by the UoA (project license 60/4007) and UCT (011/027 and 012/031).

Strains, Growth Conditions, and Infections

M. tuberculosis strain H37Rv or Beijing and *M. bovis* BCG strain Pasteur were grown on Middlebrook 7H10 agar plates containing 10% ADC (BD Biosciences) or Middlebrook 7H9 broth containing 10% ADC and 0.05% Tween 80 (Sigma). GFP-expressing *M. bovis* BCG was cultured in the presence of 10 μg/ml kanamycin (Sigma). A total of 100 colony-forming units (CFU) of *M. tuberculosis* H37Rv was administered using an inhalation exposure system (Terre Haute). For i.t. inoculations, 5×10^5 CFU *M. tuberculosis* or *M. bovis* BCG were administered to the caudal oropharynx of anesthetized mice. In some experiments, *M. bovis* BCG was opsonized with anti-BCG antiserum (Alpha Diagnostics) before i.t. challenge. Organs were homogenized in PBS containing 0.05% Triton X-100 and complete mini-EDTA-free protease inhibitors (Roche). Bacterial burdens were determined by plating onto Middlebrook 7H10 agar.

Flow Cytometric Analysis of Lung Cells

Cells were obtained from the lung by bronchio-alveolar lavage (BAL) with PBS containing 5 mM EDTA (Gibco) or by enzymatic digest with DNase (Sigma-Aldrich) and liberase (Roche). Digested tissue was passed through 70-μm and 40-μm nylon filters, and erythrocytes were lysed in Pharm Lyse solution (BD Biosciences). The following antibodies were used: CD45.2, Ly6G, CD11c, CD11b, Siglec F, CD3, CD4, CD8, CD19, Vα2, CD45.1, CD62L, CD44, CD69, CD25, IFNγ, and F4/80 (BD Biosciences or AbD Serotec). FACS was performed using an LSRII, Fortessa, or FACSAria (BD Biosciences) and analyzed using FlowJo 7.6.4. Alveolar macrophages were defined as CD11c$^+$ SiglecF$^+$, neutrophils as CD11b$^+$ Ly6G^{high}, and macrophages as CD11b^{high} F4/80$^+$.

Cytokine Assays

Tissue homogenates (above) were centrifuged to remove debris and supernatant stored at −80°C. Cytokine levels were measured using the Bio-Plex Pro Mouse 23-Plex kit (Bio-Rad) or by ELISA (BD Biosciences OptEIA and R&D Systems). Cytokine levels of tissue homogenates were normalized to sample protein concentrations.

Reporter Cell Analysis

Reporter cell analysis with NFAT-GFP expressing T hybridoma cells, co-transfected with mCLECSF8 and Fcγ, was performed as described previously (Miyake et al., 2013).

BCG Binding Experiments

For in vivo binding experiments, 1.5×10^6 CFU GFP-expressing *M. bovis* BCG was administered i.t., and BAL cells were isolated after 4 hr and analyzed by FACS. For in vitro binding experiments, BCG-GFP was added to thioglycollate-elicited macrophages (10:1) or neutrophils (1:1). In some experiments, TDM was added at 1 μg/ml. Cells were harvested and stained for CD45 and GFP positivity (indicating bacterial association) ascertained by FACS.

Genotype Analysis and Ethics Statement

We made use of a cohort of PTB patients in Indonesia (see Supplemental Experimental Procedures). Peripheral blood samples and genotyping was performed as described previously (Songane et al., 2012). All individuals recruited had signed a written informed consent. The study protocol was approved by the review boards of the University of Indonesia, the Eijkman Institute for Molecular Biology, and the Medical Ethical Committee Arnhem-Nijmegen.

Statistical Analysis

Data were analyzed using GraphPad Prism 5.04. Unpaired t test or non-parametric Mann-Whitney was applied for comparison of groups, as appropriate, and the Wilcoxon sign rank test for paired follow-up data. For genotyping analysis the Hardy-Weinberg equilibrium was checked for each SNP using the program HWE Version 1.10 (Rockefeller University). Significance was indicated by $p < 0.05$.

SUPPLEMENTAL INFORMATION

Supplemental Information includes three figures, one table, and Supplemental Experimental Procedures and can be found with this article online at http://dx.doi.org/10.1016/j.chom.2015.01.004.

AUTHOR CONTRIBUTIONS

G.J.W. and J.C.H. performed experiments with BCG. M.J.M. and J.C.H. performed experiments with Mtb. A.v.L. performed the human studies.

ACKNOWLEDGMENTS

We would like to thank S. Hardison, P. Redelinghuys, J. Taylor, C. Wallace, A. Richmond, S. Hadebe, A. Plato, F. Abbass, L. Fick, N. Allie, R. Wilkinson, K. Wilkinson, S. Cooper, D. Lang, and V. Kumar for reagents and assistance, and the animal facility staff for the care of our animals. This work was supported by the MRC (UK) and Wellcome Trust (G.D.B.); MRC (South Africa) and Sydney Brenner Fellowship (M.J.M.); Vici (M.G.N.), Vidi (R.v.C.), and Veni grants (T.S.P.) from the Netherlands Organization for Scientific Research; the Royal Netherlands Academy of Arts and Sciences (T.H.M.O.); EC FP7 projects (NEWTBVAC, ADITEC; T.H.M.O.); Carnegie Corporation and CIDRI (J.C.H.); and the University of Aberdeen (B.K.).

Received: January 7, 2014
Revised: June 6, 2014
Accepted: December 30, 2014
Published: February 11, 2015

REFERENCES

Behler, F., Steinwede, K., Balboa, L., Ueberberg, B., Maus, R., Kirchhof, G., Yamasaki, S., Welte, T., and Maus, U.A. (2012). Role of Mincle in alveolar macrophage-dependent innate immunity against mycobacterial infections in mice. J. Immunol. *189*, 3121–3129.

Berry, M.P., Graham, C.M., McNab, F.W., Xu, Z., Bloch, S.A., Oni, T., Wilkinson, K.A., Banchereau, R., Skinner, J., Wilkinson, R.J., et al. (2010). An interferon-inducible neutrophil-driven blood transcriptional signature in human tuberculosis. Nature *466*, 973–977.

Caws, M., Thwaites, G., Dunstan, S., Hawn, T.R., Lan, N.T., Thuong, N.T., Stepniewska, K., Huyen, M.N., Bang, N.D., Loc, T.H., et al. (2008). The influence of host and bacterial genotype on the development of disseminated disease with *Mycobacterium tuberculosis*. PLoS Pathog. *4*, e1000034.

Dorhoi, A., Desel, C., Yeremeev, V., Pradl, L., Brinkmann, V., Mollenkopf, H.J., Hanke, K., Gross, O., Ruland, J., and Kaufmann, S.H. (2010). The adaptor molecule CARD9 is essential for tuberculosis control. J. Exp. Med. *207*, 777–792.

Fremond, C.M., Togbe, D., Doz, E., Rose, S., Vasseur, V., Maillet, I., Jacobs, M., Ryffel, B., and Quesniaux, V.F. (2007). IL-1 receptor-mediated signal is an essential component of MyD88-dependent innate response to *Mycobacterium tuberculosis* infection. J. Immunol. *179*, 1178–1189.

Graham, L.M., Gupta, V., Schafer, G., Reid, D.M., Kimberg, M., Dennehy, K.M., Hornsell, W.G., Guler, R., Campanero-Rhodes, M.A., Palma, A.S., et al. (2012). The C-type lectin receptor CLECSF8 (CLEC4D) is expressed by

myeloid cells and triggers cellular activation through Syk kinase. J. Biol. Chem. *287*, 25964–25974.

Heitmann, L., Schoenen, H., Ehlers, S., Lang, R., and Hölscher, C. (2013). Mincle is not essential for controlling *Mycobacterium tuberculosis* infection. Immunobiology *218*, 506–516.

Ishikawa, E., Ishikawa, T., Morita, Y.S., Toyonaga, K., Yamada, H., Takeuchi, O., Kinoshita, T., Akira, S., Yoshikai, Y., and Yamasaki, S. (2009). Direct recognition of the mycobacterial glycolipid, trehalose dimycolate, by C-type lectin Mincle. J. Exp. Med. *206*, 2879–2888.

Lobato-Pascual, A., Saether, P.C., Fossum, S., Dissen, E., and Daws, M.R. (2013). Mincle, the receptor for mycobacterial cord factor, forms a functional receptor complex with MCL and FcεRI-γ. Eur. J. Immunol. *43*, 3167–3174.

Lowe, D.M., Redford, P.S., Wilkinson, R.J., O'Garra, A., and Martineau, A.R. (2012). Neutrophils in tuberculosis: friend or foe? Trends Immunol. *33*, 14–25.

Marakalala, M.J., Graham, L.M., and Brown, G.D. (2011). The role of Syk/CARD9-coupled C-type lectin receptors in immunity to *Mycobacterium tuberculosis* infections. Clin. Dev. Immunol. *2010*, 567571.

Miyake, Y., Toyonaga, K., Mori, D., Kakuta, S., Hoshino, Y., Oyamada, A., Yamada, H., Ono, K., Suyama, M., Iwakura, Y., et al. (2013). C-type lectin MCL is an FcRγ-coupled receptor that mediates the adjuvanticity of mycobacterial cord factor. Immunity *38*, 1050–1062.

Philips, J.A., and Ernst, J.D. (2012). Tuberculosis pathogenesis and immunity. Annu. Rev. Pathol. *7*, 353–384.

Schoenen, H., Bodendorfer, B., Hitchens, K., Manzanero, S., Werninghaus, K., Nimmerjahn, F., Agger, E.M., Stenger, S., Andersen, P., Ruland, J., et al. (2010). Cutting edge: Mincle is essential for recognition and adjuvanticity of the mycobacterial cord factor and its synthetic analog trehalose-dibehenate. J. Immunol. *184*, 2756–2760.

Songane, M., Kleinnijenhuis, J., Alisjahbana, B., Sahiratmadja, E., Parwati, I., Oosting, M., Plantinga, T.S., Joosten, L.A., Netea, M.G., Ottenhoff, T.H., et al. (2012). Polymorphisms in autophagy genes and susceptibility to tuberculosis. PLoS ONE *7*, e41618.

Steichen, A.L., Binstock, B.J., Mishra, B.B., and Sharma, J. (2013). C-type lectin receptor Clec4d plays a protective role in resolution of Gram-negative pneumonia. J. Leukoc. Biol. *94*, 393–398.

Tamassia, N., Zimmermann, M., Castellucci, M., Ostuni, R., Bruderek, K., Schilling, B., Brandau, S., Bazzoni, F., Natoli, G., and Cassatella, M.A. (2013). Cutting edge: an inactive chromatin configuration at the IL-10 locus in human neutrophils. J. Immunol. *190*, 1921–1925.

Yonekawa, A., Saijo, S., Hoshino, Y., Miyake, Y., Ishikawa, E., Suzukawa, M., Inoue, H., Tanaka, M., Yoneyama, M., Oh-Hora, M., et al. (2014). Dectin-2 is a direct receptor for mannose-capped lipoarabinomannan of mycobacteria. Immunity *41*, 402–413.

Zhu, L.L., Zhao, X.Q., Jiang, C., You, Y., Chen, X.P., Jiang, Y.Y., Jia, X.M., and Lin, X. (2013). C-type lectin receptors Dectin-3 and Dectin-2 form a heterodimeric pattern-recognition receptor for host defense against fungal infection. Immunity *39*, 324–334.

Cell Host & Microbe, Volume *17*

Supplemental Information

The C-Type Lectin Receptor CLECSF8/CLEC4D Is a Key Component of Anti-Mycobacterial Immunity

Gillian J. Wilson, Mohlopheni J. Marakalala, Jennifer C. Hoving, Arjan van Laarhoven, Rebecca A. Drummond, Bernhard Kerscher, Roanne Keeton, Esther van de Vosse, Tom H.M. Ottenhoff, Theo S. Plantinga, Bachti Alisjahbana, Dhirendra Govender, Gurdyal S. Besra, Mihai G. Netea, Delyth M. Reid, Janet A. Willment, Muazzam Jacobs, Sho Yamasaki, Reinout van Crevel, and Gordon D. Brown

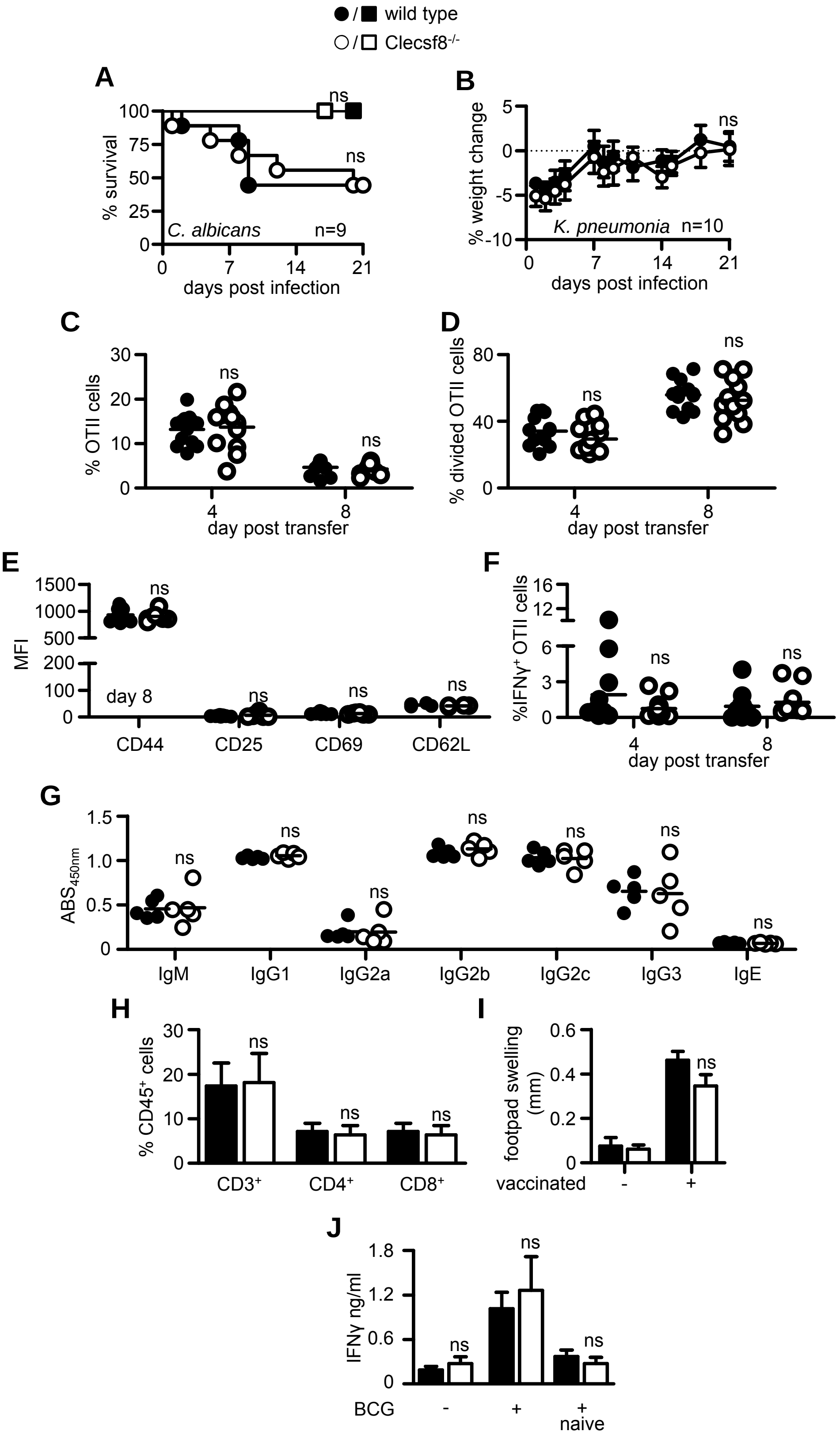

Wilson et al Supplemental Figure 1

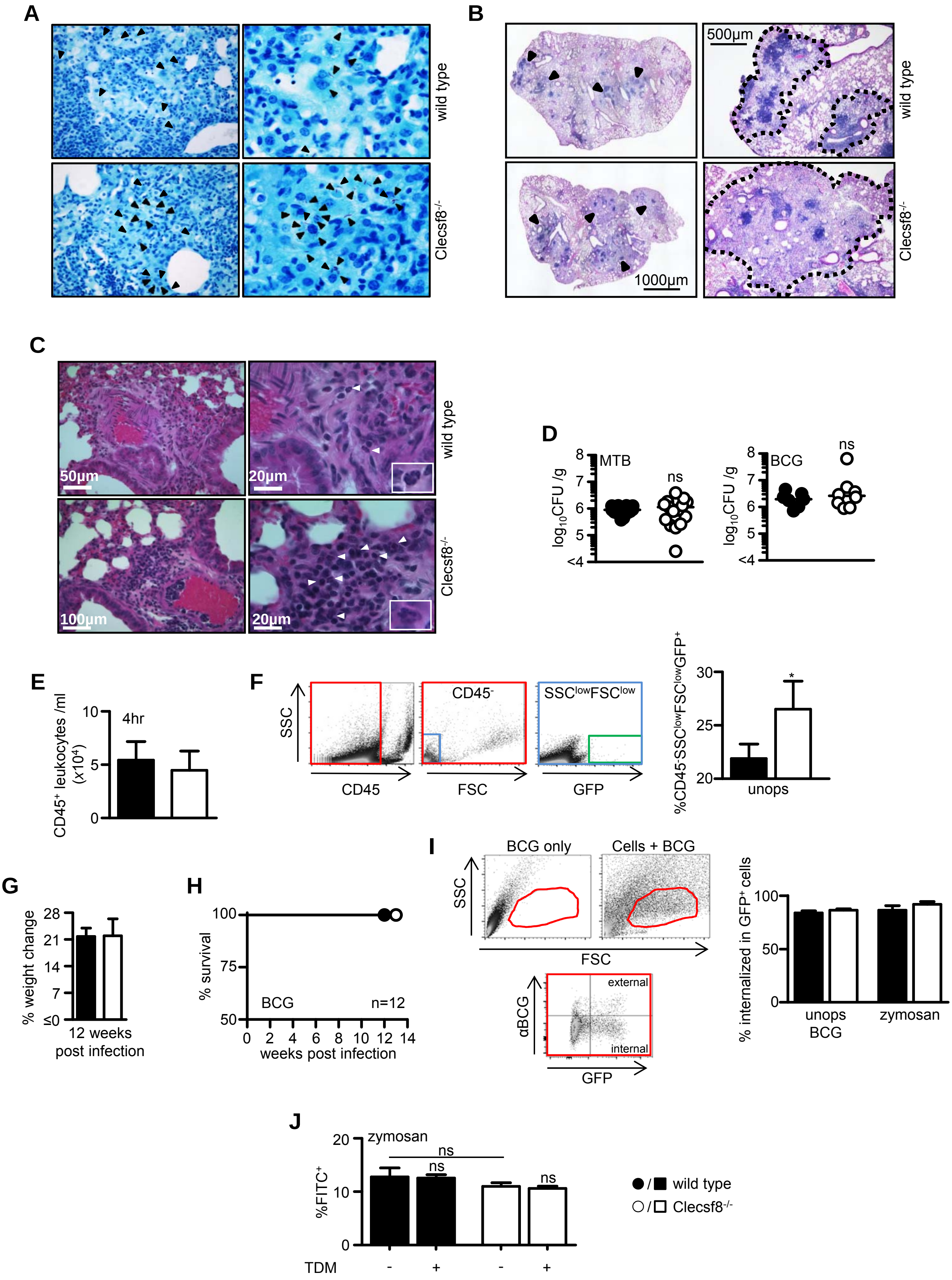
A
wild type
Clecsf8-/-
B
500μm
1000μm
wild type
Clecsf8-/-
C
50μm
20μm
100μm
20μm
wild type
Clecsf8-/-
D
MTB
ns
BCG
ns
log10CFU /g
E
4hr
CD45+ leukocytes /ml (x10^4)
F
SSC
CD45
CD45-
FSC
SSClowFSClow
GFP
%CD45-SSClowFSClowGFP+
unops
G
% weight change
12 weeks post infection
H
% survival
BCG
n=12
weeks post infection
I
BCG only
Cells + BCG
SSC
FSC
αBCG
external
internal
GFP
% internalized in GFP+ cells
unops BCG
zymosan
J
zymosan
ns
ns
ns
%FITC+
TDM
wild type
Clecsf8-/-

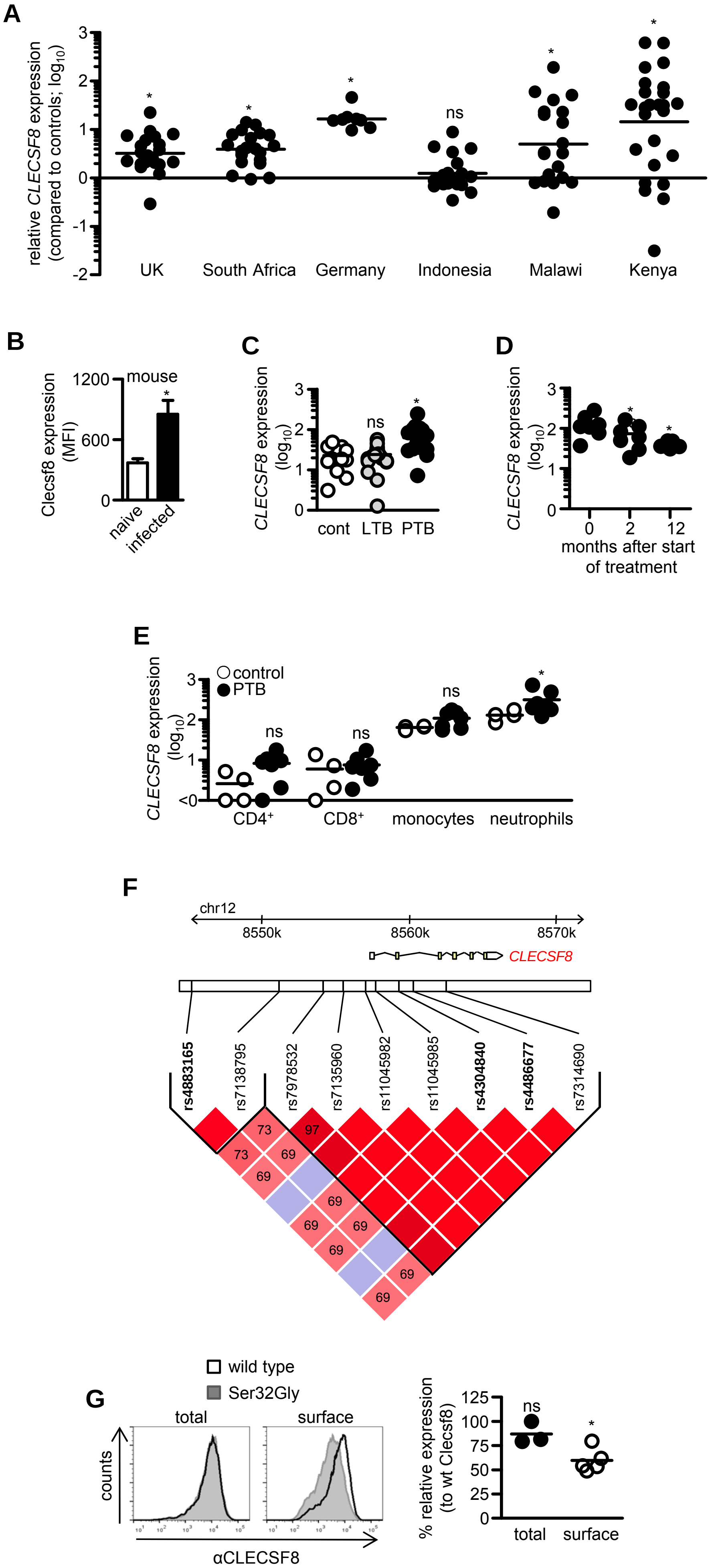

Wilson et al Supplemental Figure 3

SUPPLEMENTAL FIGURE LEGENDS

Figure S1: Clecsf8 is required for resistance to mycobacterial infection but does not impair adaptive immunity. Related to Figure 1. (A) Survival of wild-type and Clecsf8$^{-/-}$ following systemic (i.v.) infection with a high (2*x*10^5, circles) and a low (2*x*10^4, squares) dose of *Candida albicans* SC5314. (B) Change in weight of wild-type versus Clecsf8$^{-/-}$ mice following i.t. infection with 600 CFU *Klebsiella pneumoniae* strain 32. Characterization of the frequency (C), division (D), activation (E) and differentiation (F) of adoptively transferred OT.II T-cells in the draining lymph nodes of wild-type and Clecsf8$^{-/-}$ animals immunized with Ovalbumin and CFA. (G) Immunoglobulin responses in the serum of wild-type and Clecsf8$^{-/-}$ animals immunized with NP-KLH and CFA. (H) CD4$^+$ and CD8$^+$ T-cell populations in the lungs of wild-type and Clecsf8$^{-/-}$ mice 4 months after infection with 5*x*10^5 *M. bovis* BCG. (I) DTH responses in the footpad of wild-type and Clecsf8$^{-/-}$ mice 4 weeks after s.c. BCG vaccination. (J) IFN-γ recall responses following a 3-day BCG stimulation of lymph-node cells isolated from mice two weeks after infection. No cytokine response was obtained following stimulation of cells isolated from naïve animals (not shown). Values shown are from a representative experiment, except for (C, D, H - J) which are data from two pooled experiments. Each symbol (C to G) represents one mouse, and the data shown are the mean ± SD. *, $p < 0.05$; ns, not significant.

Figure S2: Clecsf8-deficiency results in higher bacterial burdens and exacerbated pulmonary inflammation. Related to Figure 2 and Figure3. (A) Ziehl-Neelsen stained lung sections from wild-type and Clecsf8$^{-/-}$ mice after 4 months of infection with *M. tuberculosis* H37Rv. Mycobacteria are indicated with arrows. (B)

Inflammatory lesion size 4 months after infection with *M. tuberculosis* H37Rv in H&E stained lung sections. (C) H&E stained lung sections at 48 hr showing accumulation of neutrophils (indicated with white arrows) after i.t. infection with a high dose ($5x10^5$) *M. tuberculosis* H37Rv. (D) Mycobacterial burdens in mice 48 h after i.t. infection with 5 x 10^5 CFU *M. tuberculosis* H37Rv or *M. bovis* BCG, as indicated. (E) Numbers of $CD45^+$ cells 4 h after i.t. inoculation of 1.5 x 10^6 CFU *M. bovis* BCG. (F) Gating strategy (control mouse shown for clarity) and percentage of non-cell associated mycobacteria (as detected in the green gate) in the lung 4 h after i.t. inoculation of 1.5 x 10^6 CFU GFP^+ *M. bovis* BCG. (G) Percentage weight change in mice 12 weeks after infection with opsonised *M. bovis* BCG. (H) Survival curve mice following i.t. infection with $5x10^5$ CFU of opsonised *M. bovis* BCG. (I) Gating strategy for flow cytometric assessment of levels of internalization of unopsonized BCG or zymosan bound to thioglycollate-elicited macrophages. Percentage internalized was defined as the percentage of APC^- (αBCG^-) GFP^+ or APC^- (FcDectin-1^-) $FITC^+$ cells versus total GFP^+ or $FITC^+$ cells. (J) Effect of TDM on *in vitro* binding of $FITC^+$ zymosan to isolated from wt or Clecsf8$^{-/-}$ mice. Shown in (D - I) are pooled data (mean ± SEM) from two independent experiments. *, $p < 0.05$.

Figure S3: Expression and polymorphisms of *CLECSF8.* Related to Table 1. (A) Relative expression of *CLECSF8* in whole blood in patients with active pulmonary TB of six cohorts, compared to the controls from the respective cohorts. (B) Clecsf8 expression on mouse $CD45^+$ leukocytes isolated from the lung 48 hr after BCG infection. (C) Expression of *CLECSF8* in whole blood in tuberculin skin test negative healthy control subjects (cont) from the UK, individuals with latent TB infection (LTB), and patients with active pulmonary TB (PTB). PTB and control data is the same as

UK cohort data in (A). (D) Expression of *CLECFS8* over time in PTB patients following initiation of TB treatment. (E) Expression of *CLECSF8* in $CD4^+$ and $CD8^+$ cells, monocytes and neutrophils of patients and healthy controls in the UK. *, $p < 0.05$. (F) Linkage disequilibrium plot of the *CLECSF8* gene region and 15kb upstream. SNPs with a prevalence of > 0.05 are shown with their linkage disequilibrium, based on data from the Han-Chinese (CHB) and Japanese (JPT) populations in Hapmap. The figure was generated with Haploview and then redrawn. Bright red indicates high level of linkage. The three SNPs that were investigated in this study (shown in bold) collectively tag the haplotypes that occur with a frequency of > 0.05 in CHB + JPT populations. (G) Example flow cytometry plots (left) and pooled analysis (right) of total and surface expression of mutated CLECSF8, relative to the wild type receptor in NIH3T3 cells expressing the Fcγ chain. Each cell line was independently generated and tested twice. *, $p < 0.05$; ns, not significant.

TABLE S1. The *CLECSF8* SNPs selected for analysis. Related to Table 1.

SNP	alleles	Minor allele frequency for the JPT & CHB population	Type
rs4883165	T / G	0.076	5'upstream of gene
rs4304840	A / G	0.129	nonsynonymous (Ser32Gly; transmembrane)
rs4486677	T / G	0.062	in intron 2

SUPPLEMENTAL EXPERIMENTAL PROCEDURES

Infections

Mice were challenged i.t. with 600 CFU *K. pneumonia* strain 32. Intravenous (i.v.) infection with low ($2x10^4$ CFU) and high ($2x10^5$ CFU) doses of *Candida albicans* SC5314 was carried out as described previously (Vautier et al., 2012). *C. albicans* hyphae were induced in 20% FCS in PBS for 3-5 h at 37°C.

Histology

Lungs were prepared for histology by fixing the large left lobe in 10% phosphate-buffered formalin and then embedding in paraffin. 5 µm-thick sections were stained with haematoxylin and eosin (H&E) for evaluation of pathologic changes and Ziehl-Neelsen (ZN) for *M. tuberculosis* detection. The sizes of all inflammatory lesions per section in infected mouse lungs were determined by automated morphometric analysis using a Nikon microscope eclipse 90i and the software NIS-Elements BR 3.1 (Nikon), as described previously (Schafer et al., 2009).

Adoptive Transfer

OT.II donor mice were culled and the lymph nodes and spleens were removed, disaggregated through 70 µM filters and white cells counted by trypan blue exclusion. $CD4^+$ cells were purified from single-cell suspensions by depleting irrelevant populations using biotin-antibody cocktail and anti-biotin micro-beads (Miltenyi Biotech). The purified $CD4^+$ cells were stained with 5 µM CFSE for 5 to 8 min at room temperature with continual rotation, and CFSE labelling was subsequently quenched by washing 2-3 times in 10% FCS in PBS. $CFSE^+CD4^+$ OT.II cells were checked by FACS for labelling efficiency and purity, which was

routinely >80%. $CFSE^+CD4^+$ OT.II cells were then injected intravenously into gender-matched recipient mice (3 to 5 x 10^6 cells per recipient) of the same background strain as the donors (C57BL/6). Mice were immunised 1 h following the adoptive transfer, with 50 µg purified ovalbumin (Hyglos GmBH) emulsified in Complete Freunds Adjuvant (Difco), delivered as 2 subcutaneous injections in the hind legs. Mice were sacrificed 4 and 8 days post-immunisation for analysis by flow cytometry. Inguinal (draining) lymph nodes were removed, made into single cell suspensions and processed for FACS, as described above standard methods. OT.II cells were defined as $CD4^+V\alpha2^+CD45.1^+$.

Immunoglobulin Assays

Mice were immunised with 100 µg NP-KLH (Biosearch Technologies) emulsified in CFA (Difco) delivered as three sub-cutaneous injections. Animals were sacrificed 10 days later, and anti-NP antibodies in the serum were measured by ELISA using specific secondary antibodies conjugated to HRP (Cambridge Biosciences).

BCG vaccination, DTH and recall responses

Mice were s.c. inoculated with 10^6 CFU *M. bovis* BCG strain Pasteur as previously described (Dorhoi et al., 2010). At 4 wk after vaccination, BCG-vaccinated and control mice were intradermally challenged with 2 µg PPD into the footpad. Footpad swelling was recorded 48 hr after challenge using a dial gauge caliper.

For recall responses, mice were infected i.t. with 5 x 10^5 CFU BCG. Draining lymph nodes were isolated after 2 weeks, disaggregated and plated at 5 x 10^5 cells per well in 96 well plates. Cells were then stimulated for 3 days with media only or

with a French-pressed BCG lysate. Cytokines were measured in the supernatant by ELISA (BD OptEIA).

Subject recruitment for genotype analysis

We previously recruited 1135 consecutive pulmonary TB patients diagnosed in two outpatient clinics and two hospitals in Jakarta and Bandung (Indonesia) from January 2001 to December 2006, for a series of genetic studies examining host susceptibility to TB ((Songane et al., 2012) and references therein). Diagnosis of pulmonary TB (PTB) was done according to WHO criteria by clinical presentation and chest radiograph examination, followed by confirmation with microscopic detection of acid-fast bacilli in ZN-stained sputum smears and positive culture of *M. tuberculosis* on 3% Ogawa medium. Patients with confirmed diagnosis of extra-pulmonary TB (n=93) and HIV-positive subjects (n=10) were excluded. During the same period, 1000 age and gender matched genetically unrelated community control subjects were selected, those with symptoms or chest X-rays suggesting possible active tuberculosis (n=48) were excluded from further analysis.

Genotyping and Selection of SNPs investigated

Using HaploView version 4.2 (Barrett et al., 2005) we accessed the publicly available HapMap Version 3 Release 2 to select SNPs for testing. As no data are available for the Indonesian population specifically, we based our selection on the data for the Han-Chinese (CHB) and Japanese (JPT) population. For *CLECSF8*, three SNPs tagged all the predicted haplotypes with frequencies of > 0.05 (See Figure S3 and Table S1).

In the current cohort, based on a disease prevalence of 262/100 000 (WHO Global tuberculosis report 2012), the power of detecting an allele that has a relative risk of 1.5 to disease, is 87% with a nominal significance of 0.05 (http://www.sph.umich.edu/csg/abecasis/cats/tour1.html).

Sample collection and genotype analysis

Peripheral blood samples were obtained by venapuncture. Genotyping was performed as described previously. In brief, genomic DNA was isolated from EDTA blood of patients and control subjects using standard methods. 5 ng of DNA was used for genotyping with multiplex assays designed using Mass ARRAY Designer Software (Sequenom) and genotypes were determined using Sequenom MALDI-TOF MS according to manufacturer’s instructions (Sequenom Inc., San Diego, CA, USA). Briefly, the SNP region was amplified by a locus-specific PCR reaction. After amplification a single base extension from a primer adjacent to the SNP was performed to introduce mass differences between alleles. This was followed by salt removal and product spotting onto a target chip with 384 patches containing matrix. MALDI-TOF MS was then used to detect mass differences and genotypes were assigned real-time using Typer 4 software (Sequenom Inc. San Diego, CA, USA). As quality control, 5% of samples were genotyped in duplicate and each 384-well plate also contained at least 8 positive and 8 negative controls, no inconsistencies were observed. For quality control purposes the genotype of at least two samples for each homozygous genotype were confirmed by sequencing using Sanger method.

Human CLECSF8 Expression Analyses

Publicly available micro-array data sets from the Gene Expression Omnibus (GEO) were analysed for the transcript of *CLECSF8* in six cohorts. Expression in whole blood of HIV-negative pulmonary TB cases was compared to expression in healthy controls or latently infected individuals from the same setting with a Mann-Whitney U test. In order to display the expression of the different cohorts in one graph, data were log-transformed and then normalised by subtracting each value with the mean value of the controls of the respective cohort, divided by the standard deviation of the controls of the respective cohort. The UK cohort in the study of Berry et al., included data on follow-up, and on cell subsets ($CD4^+$ cells, $CD8^+$ cells, monocytes and neutrophils). The cohorts used were (country; patients; GEO accession number; assay; probe):

- UK and South-Africa; adult pulmonary TB cases; GSE19491; Illumina Human HT-12 V3 BeadChip; ILMN_1808979. (Berry et al., 2010).
- Germany; adult pulmonary TB cases; GSE34608; Agilent 4 × 44-k human expression arrays; A_23_P25235. (Maertzdorf et al., 2012).
- Indonesia; adult pulmonary TB cases; GSE56153; Illumina HumanRef-8 V3 BeadChip; ILMN_1808979. (Ottenhoff, 2012).
- Malawi and Kenya: paediatric TB cases; GSE39941; HumanHT-12 v.4 Expression BeadChip; ILMN_1808979. (Anderson et al., 2014).

For quantitative trait locus (eQTL) analysis of *CLECSF8* expression in whole blood, we used data available from a meta-analysis of >5000 individuals (online available at http://genenetwork.nl/bloodeqtlbrowser) (Westra et al., 2013) and the Genotype-Tissue Expression (GTEx) consortium (Consortium, 2013), which currently includes 168 individuals (online available at http://www.gtexportal.org/).

Phagocytosis assays

Phagocytosis was measured using our previously published methodology (Herre et al., 2004). In brief, thioglycollate elicited macrophages were incubated with BCG-GFP (10:1) or zymosan at 4 °C for 1 h to allow binding, washed and incubated at 37°C 5% for 3 h to allow uptake. Extracellular BCG-GFP was stained with primary anti-BCG rabbit antiserum (Alpha diagnostics) and secondary APC-conjugated goat anti-rabbit IgG (Life Technologies). Surface-bound FITC-labelled Zymosan was stained with biotinylated Fc-Dectin-1 (Graham et al., 2006) and streptavidin-APC (Invitrogen). Phagocytosis was defined as the percentage of GFP^+ or $FITC^+$ cells with internalised (APC^-) particles. Samples kept at 4^0C to prevent internalization were used as controls.

Generation of Cell lines

Generation of the pFBneo retroviral vector containing the full length hCLECSF8 open reading frame fused to an HA-tag was described previously (Graham et al., 2012). The S32G SNP (rs4304840) was introduced by a two-step PCR protocol. Firstly, the sequences downstream and upstream of the S32G SNP were amplified separately from pFB_hCLECSF8 using a vector specific and SNP-encoding primers (pFB-neo (5'-GCCAGGTTTCCGGGCCCTCAC-3') and hCLECSF8_S32G_F (5'-TAGTTTTCATCTTACTTCTCGGTGTCTGTTTTATTGCAAG-3'); pFB-retro (5'-GGCTGCCGACCCCGGGGGTGG-3') and hCLECSF8_S32G_R (5'-CTTGCAATAAAACAGACACCGAGAAGTAAGATGAAAACTA-3'). The original pFBneo_hCLECSF8 template was digested with DpnI followed by fusion and

amplification of the two PCR products using the vector specific primers. The PCR product was cloned into pFBneo_HA and the fidelity confirmed by sequencing.

NIH 3T3 cell lines stably co-expressing FcRγ with wild-type hCLECSF8-HA, hCLECSF8_S32G-HA or empty vector control were generated by retroviral transduction as described previously (Pyz and Brown, 2011). All cell lines were generated twice and used as non-clonal populations to reduce founder effects.

Receptor expression was assessed by flow cytometry as described in the main text. Briefly, cells were blocked in FACS wash (PBS, 5 mM EDTA, 0.5% BSA, 2 mM NaN_3) containing 5% heat-inactivated rabbit serum, followed by staining with an anti-HA antibody (HA.11, clone 16B12, Covance) for 1 h at 4°C. Cells were washed twice and stained with a goat-anti-mouse PE secondary antibody (Jackson) for 30 min at 4°C. After three additional washes, cells were fixed in 1% paraformaldehyde and analysed on a BD LSRII flow cytometer. To assess the total cellular CLECSF8 content, cells were fixed in 1% paraformaldehyde followed by permeabilisation with 0.5% saponin prior to assessing receptor expression, as described above. To calculate relative expression, mean fluorescence intensities (MFI) of all samples were normalised by subtracting their respective control background MFI. Subsequently, the MFI of each cell population was divided by the average MFI of wild-type hCLECSF8 expressing cells and expressed as a percentage. Data points plotted represent the means of independent experiments.

SUPPLEMENTAL REFERENCES

Anderson, S.T., Kaforou, M., Brent, A.J., Wright, V.J., Banwell, C.M., Chagaluka, G., Crampin, A.C., Dockrell, H.M., French, N., Hamilton, M.S., *et al.* (2014). Diagnosis of childhood tuberculosis and host RNA expression in Africa. N Engl J Med *370*, 1712-1723.

Barrett, J.C., Fry, B., Maller, J., and Daly, M.J. (2005). Haploview: analysis and visualization of LD and haplotype maps. Bioinformatics *21*, 263-265.

Berry, M.P., Graham, C.M., McNab, F.W., Xu, Z., Bloch, S.A., Oni, T., Wilkinson, K.A., Banchereau, R., Skinner, J., Wilkinson, R.J., *et al.* (2010). An interferon-inducible neutrophil-driven blood transcriptional signature in human tuberculosis. Nature *466*, 973-977.

Consortium, G.T. (2013). The Genotype-Tissue Expression (GTEx) project. Nat Genet *45*, 580-585.

Dorhoi, A., Desel, C., Yeremeev, V., Pradl, L., Brinkmann, V., Mollenkopf, H.J., Hanke, K., Gross, O., Ruland, J., and Kaufmann, S.H. (2010). The adaptor molecule CARD9 is essential for tuberculosis control. J Exp Med *207*, 777-792.

Graham, L.M., Gupta, V., Schafer, G., Reid, D.M., Kimberg, M., Dennehy, K.M., Horsnell, W.G., Guler, R., Campanero-Rhodes, M.A., Palma, A.S., *et al.* (2012). The C-type lectin receptor CLECSF8(CLEC4D) is expressed by myeloid cells and triggers cellular activation through SYK kinase. J Biol Chem *287*, 25964-25974.

Graham, L.M., Tsoni, S.V., Willment, J.A., Williams, D.L., Taylor, P.R., Gordon, S., Dennehy, K., and Brown, G.D. (2006). Soluble Dectin-1 as a tool to detect beta-glucans. J Immunol Methods *314*, 164-169.

Herre, J., Marshall, A.J., Caron, E., Edwards, A.D., Williams, D.L., Schweighoffer, E., Tybulewicz, V.L., Reis e Sousa, C., Gordon, S., and Brown, G.D. (2004). Dectin-1 utilizes novel mechanisms for yeast phagocytosis in macrophages. Blood *104*, 4038-4045.

Maertzdorf, J., Weiner, J., 3rd, Mollenkopf, H.J., Bauer, T., Prasse, A., Muller-Quernheim, J., and Kaufmann, S.H. (2012). Common patterns and disease-related signatures in tuberculosis and sarcoidosis. Proc Natl Acad Sci U S A *109*, 7853-7858.

Ottenhoff, T.H. (2012). New pathways of protective and pathological host defense to mycobacteria. Trends Microbiol *20*, 419-428.

Pyz, E., and Brown, G.D. (2011). Screening for ligands of C-type lectin-like receptors. Methods Mol Biol *748*, 1-19.

Schafer, G., Guler, R., Murray, G., Brombacher, F., and Brown, G.D. (2009). The role of scavenger receptor B1 in infection with Mycobacterium tuberculosis in a murine model. PLoS ONE *4*, e8448.

Songane, M., Kleinnijenhuis, J., Alisjahbana, B., Sahiratmadja, E., Parwati, I., Oosting, M., Plantinga, T.S., Joosten, L.A., Netea, M.G., Ottenhoff, T.H.*, et al.* (2012). Polymorphisms in autophagy genes and susceptibility to tuberculosis. PLoS ONE *7*, e41618.

Vautier, S., Drummond, R.A., Redelinghuys, P., Murray, G.I., MacCallum, D.M., and Brown, G.D. (2012). Dectin-1 is not required for controlling Candida albicans colonization of the gastrointestinal tract. Infect Immun *80*, 4216-4222.

Westra, H.J., Peters, M.J., Esko, T., Yaghootkar, H., Schurmann, C., Kettunen, J., Christiansen, M.W., Fairfax, B.P., Schramm, K., Powell, J.E.*, et al.* (2013). Systematic identification of trans eQTLs as putative drivers of known disease associations. Nat Genet *45*, 1238-1243.
